# Genome-wide association study of classical Hodgkin lymphoma identifies key regulators of disease susceptibility

**DOI:** 10.1038/s41467-017-00320-1

**Published:** 2017-12-01

**Authors:** Amit Sud, Hauke Thomsen, Philip J. Law, Asta Försti, Miguel Inacio da Silva Filho, Amy Holroyd, Peter Broderick, Giulia Orlando, Oleg Lenive, Lauren Wright, Rosie Cooke, Douglas Easton, Paul Pharoah, Alison Dunning, Julian Peto, Federico Canzian, Rosalind Eeles, ZSofia Kote-Jarai, Kenneth Muir, Nora Pashayan, The PRACTICAL consortium, Brian E. Henderson, Christopher A. Haiman, Sara Benlloch, Fredrick R. Schumacher, Ali Amin Al Olama, Sonja I. Berndt, David V. Conti, Fredrik Wiklund, Stephen Chanock, Victoria L. Stevens, Catherine M. Tangen, Jyotsna Batra, Judith Clements, Henrik Gronberg, Johanna Schleutker, Demetrius Albanes, Stephanie Weinstein, Alicja Wolk, Catharine West, Lorelei Mucci, Géraldine Cancel-Tassin, Stella Koutros, Karina Dalsgaard Sorensen, Lovise Maehle, David E. Neal, Ruth C. Travis, Robert J. Hamilton, Sue Ann Ingles, Barry Rosenstein, Yong-Jie Lu, Graham G. Giles, Adam S. Kibel, Ana Vega, Manolis Kogevinas, Kathryn L. Penney, Jong Y. Park, Janet L. Stanford, Cezary Cybulski, Børge G. Nordestgaard, Hermann Brenner, Christiane Maier, Jeri Kim, Esther M. John, Manuel R. Teixeira, Susan L. Neuhausen, Kim De Ruyck, Azad Razack, Lisa F. Newcomb, Davor Lessel, Radka Kaneva, Nawaid Usmani, Frank Claessens, Paul A. Townsend, Manuela Gago-Dominguez, Monique J. Roobol, Florence Menegaux, Per Hoffmann, Markus M. Nöthen, Karl-Heinz Jöckel, Elke Pogge von Strandmann, Tracy Lightfoot, Eleanor Kane, Eve Roman, Annette Lake, Dorothy Montgomery, Ruth F. Jarrett, Anthony J. Swerdlow, Andreas Engert, Nick Orr, Kari Hemminki, Richard S. Houlston

**Affiliations:** 1 Division of Genetics and Epidemiology, The Institute of Cancer Research, London, SW7 3RP UK; 2 Division of Molecular Genetic Epidemiology, German Cancer Research Centre, Heidelberg, 69120 Germany; 3 Centre for Primary Health Care Research, Lund University, Malmö, 221 00 Sweden; 4 Centre for Cancer Genetic Epidemiology, Department of Oncology, University of Cambridge, Cambridge, CB1 8RN UK; 5 Centre for Cancer Genetic Epidemiology, Department of Public Health and Primary Care, University of Cambridge, Cambridge, CB1 8RN UK; 6 Department of Non-Communicable Disease Epidemiology, London School of Hygiene and Tropical Medicine, London, WC1E 7HT UK; 7 Genomic Epidemiology Group, German Cancer Research Center (DKFZ), Heidelberg, 69120 Germany; 8 Royal Marsden NHS Foundation Trust, London, SM2 5NG UK; 9 Institute of Population Health, University of Manchester, Manchester, M1 3BB UK; 10 Division of Health Sciences, Warwick Medical School, Warwick University, Warwick, CV4 7AL UK; 11 Department of Applied Health Research, University College London, London, WC1E 7HB UK; 12 Department of Biomedicine, Division of Medical Genetics, University of Basel, Basel, 4031 Switzerland; 13 Institute of Human Genetics, University of Bonn, Bonn, 53127 Germany; 14 Department of Genomics, Life & Brain Center, University of Bonn, Bonn, 53127 Germany; 15 University of Duisburg–Essen, Essen, 47057 Germany; 16 Department of Internal Medicine, University Hospital of Cologne, Cologne, 50937 Germany; 17 Department of Health Sciences, University of York, York, YO10 5DD UK; 18 MRC University of Glasgow Centre for Virus Research, Glasgow, G61 1QH UK; 19 Division of Breast Cancer Research, The Institute of Cancer Research, London, SW7 3RP UK; 20 Division of Molecular Pathology, The Institute of Cancer Research, London, SW7 3RP UK; 21 Department of Preventive Medicine, Keck School of Medicine, University of Southern California/Norris Comprehensive Cancer Center, Los Angeles, CA 90033 USA; 22 Department of Epidemiology and Biostatistics, Case Western Reserve University, Cleveland, OH 44106 USA; 23 Seidman Cancer Center, University Hospitals, Cleveland, OH 44106 USA; 24 Department of Clinical Neurosciences, University of Cambridge, Cambridge, CB2 1TN UK; 25 Division of Cancer Epidemiology and Genetics, National Cancer Institute, NIH, Bethesda, MD 20892 USA; 26 Department of Medical Epidemiology and Biostatistics, Karolinska Institute, SE-171 77 Stockholm, Sweden; 27 Epidemiology Research Program, American Cancer Society, 250 Williams Street, Atlanta, Georgia 30303 USA; 28 SWOG Statistical Center, Fred Hutchinson Cancer Research Center, Seattle, WA 98109 USA; 29 Australian Prostate Cancer Research Centre-Qld, Institute of Health and Biomedical Innovation and School of Biomedical Science, Queensland University of Technology, Brisbane, QLD 4059 Australia; 30 Translational Research Institute, Brisbane, QLD 4102 Australia; 31 Department of Medical Biochemistry and Genetics, Institute of Biomedicine, University of Turku, FI-20520 Turku, Finland; 32 Tyks Microbiology and Genetics, Department of Medical Genetics, Turku University Hospital, FI-20520 Turku, Finland; 33 BioMediTech, University of Tampere, Tampere, 33100 Finland; 34 Division of Nutritional Epidemiology, Institute of Environmental Medicine, Karolinska Institutet, SE-171 77 Stockholm, Sweden; 35 Institute of Cancer Sciences, University of Manchester, Manchester Academic Health Science Centre, Radiotherapy Related Research, The Christie Hospital NHS Foundation Trust, Manchester, M13 9NT UK; 36 Department of Epidemiology, Harvard School of Public Health, Boston, MA 02115 USA; 37 CeRePP, Pitie-Salpetriere Hospital, 75013 Paris, France; 38 UPMC Univ Paris 06, GRC N°5 ONCOTYPE-URO, CeRePP, Tenon Hospital, 75020 Paris, France; 39 Department of Molecular Medicine, Aarhus University Hospital, 8000 Aarhus C, Denmark; 40 Department of Clinical Medicine, Aarhus University, 8000 Aarhus C, Denmark; 41 Department of Medical Genetics, Oslo University Hospital, N-0424 Oslo, Norway; 42 Department of Oncology, Addenbrooke’s Hospital, University of Cambridge, Cambridge, CB2 0QQ UK; 43 Cancer Research UK, Cambridge Research Institute, Li Ka Shing Centre, Cambridge, CB2 0RE UK; 44 Cancer Epidemiology, Nuffield Department of Population Health, University of Oxford, Oxford, OX3 7LF UK; 45 Dept. of Surgical Oncology, Princess Margaret Cancer Centre, Toronto, ON M5G 2M9 Canada; 46 Department of Radiation Oncology, Icahn School of Medicine at Mount Sinai, New York, NY 10029 USA; 47 Department of Genetics and Genomic Sciences, Icahn School of Medicine at Mount Sinai, New York,, NY 10029 USA; 48 Centre for Molecular Oncology, Barts Cancer Institute, Queen Mary University of London, John Vane Science Centre, London, EC1M 6BQ UK; 49 Cancer Epidemiology Centre, The Cancer Council Victoria, Melbourne, VIC 3004 Australia; 50 Centre for Epidemiology and Biostatistics, Melbourne School of Population and Global Health, The University of Melbourne, Melbourne, VIC 3053 Australia; 51 Division of Urologic Surgery, Brigham and Womens Hospital, Boston, MA 02115 USA; 52 Fundación Pública Galega de Medicina Xenómica-SERGAS, Grupo de Medicina Xenómica, CIBERER, IDIS, 15782 Santiago de Compostela, Spain; 53 Centre for Research in Environmental Epidemiology (CREAL), Barcelona Institute for Global Health (ISGlobal), 60803 Barcelona, Spain; 54 CIBER Epidemiología y Salud Pública (CIBERESP), 28029 Madrid, Spain; 55 IMIM (Hospital del Mar Research Institute), 08003 Barcelona, Spain; 56 Universitat Pompeu Fabra (UPF), 08002 Barcelona, Spain; 57 Channing Division of Network Medicine, Department of Medicine, Brigham and Women’s Hospital/Harvard Medical School, Boston, MA 02115 USA; 58 Department of Cancer Epidemiology, Moffitt Cancer Center, Tampa, FL 33612 USA; 59 Division of Public Health Sciences, Fred Hutchinson Cancer Research Center, Seattle, WA 98109 USA; 60 Department of Epidemiology, School of Public Health, University of Washington, Seattle, WA 98195 USA; 61 International Hereditary Cancer Center, Department of Genetics and Pathology, Pomeranian Medical University, 70-001 Szczecin, Poland; 62 Faculty of Health and Medical Sciences, University of Copenhagen, 1165 Copenhagen, Denmark; 63 Department of Clinical Biochemistry, Herlev and Gentofte Hospital, University Hospital, 2900 Copenhagen, Denmark; 64 Division of Clinical Epidemiology and Aging Research, German Cancer Research Center (DKFZ), 69120 Heidelberg, Germany; 65 German Cancer Consortium (DKTK), German Cancer Research Center (DKFZ), 69120 Heidelberg, Germany; 66 Division of Preventive Oncology, German Cancer Research Center (DKFZ) and National Center for Tumor Diseases (NCT), 69120 Heidelberg, Germany; 67 Institute for Human Genetics, University Hospital Ulm, 89081 Ulm, Germany; 68 Department of Genitourinary Medical Oncology, The University of Texas M. D. Anderson Cancer Center, Houston, TX 77030 USA; 69 Cancer Prevention Institute of California, Fremont, CA 94538 USA; 70 Department of Health Research & Policy (Epidemiology) and Stanford Cancer Institute, Stanford University School of Medicine, Stanford, CA 94305 USA; 71 Department of Genetics, Portuguese Oncology Institute of Porto, 4200-072 Porto, Portugal; 72 Biomedical Sciences Institute (ICBAS), University of Porto, 4200-072 Porto, Portugal; 73 Department of Population Sciences, Beckman Research Institute of the City of Hope, Duarte, CA 91016 USA; 74 Faculty of Medicine and Health Sciences, Basic Medical Sciences, Ghent University, 9000 Ghent, Belgium; 75 Department of Surgery, Faculty of Medicine, University of Malaya, 50603 Kuala Lumpur, Malaysia; 76 Department of Urology, University of Washington, Seattle, WA 98105 USA; 77 Institute of Human Genetics, University Medical Center Hamburg-Eppendorf, 20246 Hamburg, Germany; 78 Molecular Medicine Center, Department of Medical Chemistry and Biochemistry, Medical University, 1431 Sofia, Bulgaria; 79 Department of Oncology, Cross Cancer Institute, University of Alberta, Edmonton, AB T6G 2R3 Canada; 80 Division of Radiation Oncology, Cross Cancer Institute, Edmonton, AB T6G 1Z2 Canada; 81 Molecular Endocrinology Laboratory, Department of Cellular and Molecular Medicine, KU Leuven, 3000 Leuven, Belgium; 82 Institute of Cancer Sciences, Manchester Cancer Research Centre, University of Manchester, Manchester Academic Health Science Centre, St Mary’s Hospital, Manchester, M13 9WL UK; 83 Genomic Medicine Group, Galician Foundation of Genomic Medicine, Instituto de Investigacion Sanitaria de Santiago de Compostela (IDIS), Complejo Hospitalario Universitario de Santiago, Servicio Galego de Saúde, SERGAS, 15706 Santiago De Compostela, Spain; 84 University of California San Diego, Moores Cancer Center, La Jolla, CA 92093 USA; 85 Department of Urology, Erasmus University Medical Center, 3015 CE Rotterdam, The Netherlands; 86 Cancer & Environment Group, Center for Research in Epidemiology and Population Health (CESP), INSERM, University Paris-Sud, University Paris-Saclay, F-94805 Villejuif, France

## Abstract

Several susceptibility loci for classical Hodgkin lymphoma have been reported. However, much of the heritable risk is unknown. Here, we perform a meta-analysis of two existing genome-wide association studies, a new genome-wide association study, and replication totalling 5,314 cases and 16,749 controls. We identify risk loci for all classical Hodgkin lymphoma at 6q22.33 (rs9482849*, P* = 1.52 × 10^−8^) and for nodular sclerosis Hodgkin lymphoma at 3q28 (rs4459895, *P* = 9.43 × 10^−17^), 6q23.3 (rs6928977, *P* = 4.62 × 10^−11^), 10p14 (rs3781093, *P* = 9.49 × 10^−13^), 13q34 (rs112998813, *P* = 4.58 × 10^−8^) and 16p13.13 (rs34972832*, P* = 2.12 × 10^−8^). Additionally, independent loci within the HLA region are observed for nodular sclerosis Hodgkin lymphoma (rs9269081, HLA-DPB1*03:01, Val86 in HLA-DRB1) and mixed cellularity Hodgkin lymphoma (rs1633096, rs13196329, Val86 in HLA-DRB1). The new and established risk loci localise to areas of active chromatin and show an over-representation of transcription factor binding for determinants of B-cell development and immune response.

## Abstract (web summary)

Classical Hodgkin lymphoma is a cancer that originates in lymph nodes. Little is known about its genetic susceptibility. Here, the authors combined existing and new genome-wide association studies to identify risk loci for classical Hodgkin lymphoma at 6q22.33, and nodular sclerosis Hodgkin lymphoma at 3q28, 6q23.3, 10p14, 13q34, 16p13.13.

## Introduction

Classical Hodgkin lymphoma (cHL) is a lymphoid malignancy of germinal centre (GC) B-cell origin^1^, which is characterised by Hodgkin and Reed–Sternberg (HRS) cells with a dominant background population of reactive inflammatory cells^1^. Of the four major subtypes of cHL, nodular sclerosis Hodgkin lymphoma (NSHL) and mixed cellularity Hodgkin lymphoma (MCHL) account for 65% and 20% of cHL, respectively^2^. While Epstein–Barr virus (EBV) infection is causally associated with a subset of cHL cases, proportionally higher in MCHL, no other environmental factor has thus far been robustly linked to cHL risk^3^.

Evidence for inherited genetic influence on susceptibility to cHL is provided by the familial risk and the high concordance between monozygotic twins^4, 5^. A strong HLA association for cHL risk is well established; however, our understanding of cHL heritability has been transformed by recent genome-wide association studies (GWAS), which have identified single-nucleotide polymorphisms (SNPs) at seven non-HLA loci influencing risk^6–9^. Although projections indicate that additional risk variants for cHL can be discovered by GWAS^10^, the statistical power of published studies is limited.

To gain a more comprehensive insight into cHL predisposition, we performed a meta-analysis of two previous GWAS^7, 8^ and a new GWAS, thereby more than doubling study power to discover risk SNPs. With replication, our study has allowed us to identify six new non-HLA risk loci. Additionally, by conducting region-specific imputation we have defined the specific HLA associations underlying NSHL and MCHL risk.

## Results

### Association analysis

We analysed GWAS data from three studies of European ancestry: a new GWAS from the UK National Study of Hodgkin Lymphoma Genetics (NSHLG) and two previously reported GWAS (Supplementary Table 1)^7, 8^. After quality control the three studies provided SNP genotypes on 3,077 cases and 13,680 controls (Supplementary Tables 2, 3, 4; Supplementary Fig. 1). To increase genomic resolution, we imputed >10 million SNPs using the 1000 Genomes Project and the UK10K data as reference^11, 12^. Quantile–quantile (Q–Q) plots for SNPs with minor allele frequency (MAF) > 0.05% post imputation did not show evidence of substantive over-dispersion (*λ* = 1.03–1.09; Supplementary Fig. 2). An overview of the analysis strategy is outlined in Supplementary Fig. 3. Meta-analysing the association test results from the three GWAS into a joint discovery set, we calculated joint odds ratios and 95% confidence intervals for each SNP and associated per-allele *P*-value for all cHL, NSHL and MCHL cases vs. controls (Supplementary Fig. 4). In this analysis, associations for the established non-HLA risk loci at 2p16.1, 3p24.1, 5q31.1, 6q23.3, 8q24.21, 10p14 and 19p13.3 were consistent in direction and magnitude of effect with previously reported studies (Supplementary Fig. 4; Supplementary Table 5**)**^6–8^.

We sought validation of association SNPs with a *P*-value from the meta-analysis under a fixed-effects model at *P* < 1.0 × 10^−7^ and *P* < 1.0 × 10^−6^ for loci not previously associated with cHL and NSHL risk, respectively, by genotyping two additional independent series (Supplementary Table 1), totalling 2,237 cases and 3,069 controls (Table 1; Supplementary Table 6). Where the strongest signal was provided by an imputed SNP, we confirmed the fidelity of imputation by genotyping (Supplementary Table 7). In the combined meta-analysis, we identified genome-wide significant associations for cHL (Table 1; Supplementary Tables 8 and 9), at 3q28 (rs4459895, *P* = 4.45 × 10^−18^), 6q22.33 (rs9482849, *P* = 1.52 × 10^−8^), 6q23.3 (rs6928977, *P* = 1.24 × 10^−10^) and 10p14 (rs3781093, *P* = 4.91 × 10^−12^), which were predominantly driven by an association with NSHL. The rs6928977 association is independent of the previously identified association at 6q23.3 marked by rs9402684 (Supplementary Table 5); respective conditional *P*-values, *P* = 1.28 × 10^−8^ and *P* = 9.80 × 10^−6^ (pairwise LD metrics *r*^2^ = 0.002, *D*’ = 0.007)^8^. Furthermore, the rs3781093 association is independent of the previously identified 10p14 association marked by rs2388486 (Supplementary Table 5); respective conditional *P*-values are *P* = 3.38 × 10^−8^ and *P* = 1.32 × 10^−12^ (pairwise LD metrics *r*^2^ = 0.002, *D*’ = 0.27)^7^. For NSHL we identified two new associations at 13q34 (rs112998813, *P* = 4.58 × 10^−8^) and 16p13.13 (rs34972832, *P* = 2.12 × 10^−8^, Table 1).

**Table 1:** Summary results for newly identified risk loci

	Nearest gene^a^	Risk allele (frequency)		Discovery GWAS meta-analysis		UK Replication 1		UK Replication 2		Meta-analysis			
			Position (hg19, bp)	*P*-value	OR (95% CI)	*P*-value	OR (95% CI)	*P*-value	OR (95% CI)	*P*-value	OR (95% CI)	*I*^2^ (%)	*P*_het_
3q28, rs4459895	*LPP*	A (0.20)	187954414										
cHL				4.16 × 10^−10^	1.27 (1.18–1.36)	6.85 × 10^−9^	1.44 (1.27–1.63)	0.02	1.26 (1.04–1.52)	4.45 × 10^−18^	1.30 (1.23–1.38)	13	0.33
NSHL				9.16 × 10^−9^	1.37 (1.23–1.53)	1.37 × 10^−8^	1.43 (1.26–1.62)	0.04	1.30 (1.02–1.66)	9.43 × 10^−17^	1.39 (1.28–1.50)	0	0.93
MCHL				0.92	1.04 (0.91–1.19)			0.98	1.00 (0.68–1.47)	0.55	1.04 (0.92–1.19)	0	0.82
6q22.33, rs9482849	*PTPRK*	C (0.17)	128288536										
cHL				5.02 × 10^−8^	1.24 (1.15–1.35)	0.13	1.11 (0.97–1.27)	0.13	1.17 (0.95–1.43)	1.52 × 10^−8^	1.20 (1.13–1.28)	3	0.39
NSHL				2.91 × 10^−6^	1.32 (1.17–1.48)	0.17	1.10 (0.96–1.25)	0.20	1.19 (0.91–1.54)	4.13 × 10^−6^	1.21 (1.12–1.33)	10	0.35
MCHL				0.17	1.11 (0.96–1.28)			0.78	1.06 (0.70–1.61)	0.16	1.10 (0.96–1.26)	0	0.97
6q23.3, rs6928977	*AHI1*	G (0.57)	135626348										
cHL				1.66 × 10^−8^	1.18 (1.12–1.26)	0.01	1.14 (1.03–1.26)	0.05	1.16 (1.00–1.34)	1.24 × 10^−10^	1.17 (1.12–1.23)	0	0.85
NSHL				9.34 × 10^−10^	1.31 (1.20–1.42)	0.03	1.12 (1.01–1.24)	0.01	1.30 (1.06–1.58)	4.62 × 10^−11^	1.23 (1.16–1.31)	26	0.25
MCHL				0.24	1.06 (0.96–1.18)			0.69	1.06 (0.79–1.42)	0.22	1.06 (0.96–1.17)	0	0.22
10p14, rs3781093	*GATA3*	T (0.88)	8101927										
cHL				4.89 × 10^−12^	1.35 (1.23–1.47)	4.00 × 10^−4^	1.32 (1.25–1.44)	0.11	1.21 (0.96–1.52)	4.91 × 10^−12^	1.28 (1.19–1.37)	64	0.01
NSHL				9.16 × 10^−12^	1.53 (1.36–1.75)	2.00 × 10^−4^	1.44 (1.31–1.61)	0.64	0.92 (0.68–1.26)	9.49 × 10^−13^	1.39 (1.28–1.53)	61	0.06
MCHL				0.03	1.18 (1.02–1.36)			0.05	1.56 (1.02–2.40)	0.16	0.91 (0.79–1.04)	73	0.03
13q34, rs112998813	*UPF3A*	C (0.08)	115059729										
cHL				3.63 × 10^−3^	1.19 (1.06–1.33)	0.03	1.23 (1.03–1.47)	0.43	1.12 (0.84–1.50)	2.70 × 10^−4^	1.19 (1.08–1.30)	13	0.32
NSHL				8.43 × 10^−8^	1.58 (1.34–1.88)	0.03	1.22 (1.02–1.47)	0.28	1.23 (0.85–1.78)	4.58 × 10^−8^	1.39 (1.23–1.56)	27	0.24
MCHL				0.92	0.99 (0.80–1.22)			0.27	1.35 (0.80–2.23)	0.75	1.03 (0.85–1.25)	0	0.56
16p13.13, rs34972832	*CLEC16A*	A (0.18)	11198938										
cHL				1.45 × 10^−4^	1.15 (1.07–1.23)	6.34 × 10^−3^	1.18 (1.05–1.34)	0.10	1.17 (0.97–1.42)	8.03 × 10^−7^	1.16 (1.09–1.23)	6	0.37
NSHL				7.47 × 10^−7^	1.24 (1.15–1.34)	6.53 × 10^−3^	1.30 (1.17–1.45)	0.28	1.15 (0.89–1.50)	2.12 × 10^−8^	1.24 (1.15–1.34)	37	0.18
MCHL				0.65	0.97 (0.85–1.11)			0.91	1.02 (0.69–1.52)	0.70	0.98 (0.86–1.10)	0	0.94

The risk allele is the allele corresponding to the estimated odds ratio. Frequency of the risk allele is from the CEU population from 1000 Genomes Project

*cHL* classical Hodgkin lymphoma, *NSHL* nodular sclerosis Hodgkin lymphoma, *MCHL* mixed cellularity Hodgkin lymphoma, *bp* base pair, *OR* odds ratio, *CI* confidence interval, *I*^2^ proportion of the total variation due to heterogeneity

*I*^2^ value ≥ 75% is considered to be characteristic of large heterogeneity

^a^Nearest gene may not be the functional gene

### Relationship between the new risk SNPs and phenotype

A hallmark of cHL epidemiology is the bimodal age-specific incidence and it has been argued that the disease in young adults and older adults is aetiologically different; in particular there is a low prevalence of EBV-positive disease in NSHL patients aged 16–35^3^. Case-only analysis did not provide evidence of sex differences at newly identified risk SNPs (Supplementary Table 10) or a relationship between age in the NSHL subgroup. Albeit not significant after correction for multiple testing, we observed an association between EBV-positive disease and cHL at 6q23.3 in 796 cases analysed (rs6928977, *P* = 0.03, Supplementary Table 10).

### Biological inference

**Fig. 1:**
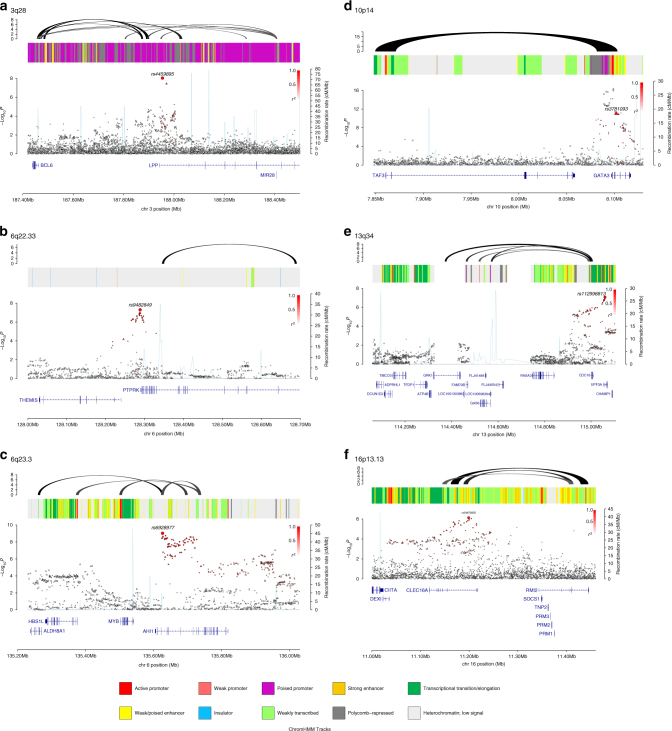
Regional plots of association results and recombination rates for the newly identified classical Hodgkin lymphoma (NSHL) risk loci. Results for **a** 3q28 (rs4459895) and nodular sclerosis Hodgkin lymphoma (NSHL) risk, **b** 6q22.33 (rs9482849) and classical Hodgkin lymphoma (cHL) risk, **c** 6q23.3 (rs6928977) and NSHL risk, **d** 10p14 (rs3781093) and NSHL risk, **e** 13q34 (rs112998813) and NSHL risk, and **f** 16p13.13 (rs34972832) and NSHL risk. Plots show association results of both genotyped (*triangles*) and imputed (*circles*) single-nucleotide polymorphisms (SNPs) in the genome-wide association study samples and recombination rates. −log_10_ *P-*values (*y*-axes) of the SNPs are shown according to their chromosomal positions (*x*-axes). The sentinel SNP in each combined analysis is shown as a large *circle* or *triangle* and is labelled by its rsID. The colour intensity of each symbol reflects the extent of LD with the top SNP, *white* (*r*^2^ = 0) through to *dark red* (*r*^2^ = 1.0). Genetic recombination rates, estimated using 1000 Genomes Project samples, are shown with a *light blue line*. Physical positions are based on NCBI build 37 of the human genome. Also shown are the relative positions of genes and transcripts mapping to the region of association. Genes have been redrawn to show their relative positions; therefore maps are not to physical scale. The middle track represents the chromatin state segmentation track (ChromHMM) for lymphoblastoid cells using data from the HapMap ENCODE Project. The top track represents capture Hi-C promoter contacts in GM12878 cells. The colour intensity of each contact reflects the interaction score

Five of the six new risk SNPs localise in or near genes which have either been previously implicated in the development of cHL or have established roles in B-cell development and are therefore strong candidates for cHL susceptibility. Specifically, the 6q22.33 association marked by rs9482849 maps intergenically to *THEMIS* (thymocyte-expressed molecule involved in selection) and *PTPRK* (receptor-type tyrosine protein phosphatase kappa) (Fig. 1). Downregulation of *PTPRK* by the EBV-encoded EBNA1 contributes to the growth and survival of HRS cells^13^. The 6q23.3 association defined by rs6928977 localises to intron 3 of *AHI1* (abelson helper integration site-1) (Fig. 1) which has been implicated in the development of both B- and T-cell lymphoma^14, 15^. The 13q34 association marked by rs112998813 is located in intron 5 of *UPF3A* (Fig. 1), a regulator of nonsense transcripts^16^. The LD region of association also harbours *CDC16* (cell division cycle protein 16). CDC16, a subunit of the anaphase-promoting complex^17^, targets cell cycle regulatory proteins for proteasome degradation, thereby allowing cell cycle progression, and is downregulated in HRS cells^18^. At 16p13.13, the rs34972832 association for NSHL maps to intron 18 of *CLEC16A* (C-type lectin domain family 1, Fig. 1) whose loss of function affects both B-cell number and function^19^. The 10p14 association marked by rs3781093 maps intronic of *GATA3* (Fig. 1). Transcriptional repression of *GATA3* is essential for early B-cell commitment, and aberrant *GATA3* expression has been observed in HRS cells^20, 21^. Intriguingly, the rs3781093 risk allele for NSHL has previously been demonstrated to be protective for paediatric B-cell acute lymphoblastic leukaemia (ALL)^22^.

To the extent that they have been deciphered, many GWAS risk loci map to non-coding regions of the genome and influence gene regulation. Hence, to gain insight into the biological mechanisms for the associations of the newly identified risk SNPs, we interrogated publicly accessible expression data on lymphoblastoid cell lines (LCLs)^23, 24^. We used the summary data-based Mendelian randomisation (SMR) analysis to test for pleiotropy between GWAS signal and *cis*-expression quantitative trait (eQTL) for genes within 1 Mb of the sentinel SNP at each locus to identify a causal relationship^25^. At 6q23.3 and 10p14, significant eQTLs were observed with *AHI1* (*P*_SMR_ = 8.63 × 10^−6^; Supplementary Table 11 and Supplementary Fig. 5) and *GATA3* (*P*_SMR_ = 4.70 × 10^−8^; Supplementary Table 11 and Supplementary Fig. 5).

Since spatial proximity between specific genomic regions and chromatin looping interactions are central for the regulation of gene expression, we identified patterns of chromatin interactions at candidate causal SNPs by analysing promoter capture Hi-C data on GM12878 as a source of B-cell information^26^. Looping chromatin interactions were shown at 3q28 (rs4459895), 6q23.3 (rs6928977), 10p14 (rs3781093) and 16p13.13 (rs34972832). While no significant eQTL was shown for these chromatin looping interactions they involved a number of genes with biological relevance to cHL development (Fig. 1). At 3q28, the looping interaction implicates *BCL6* and mir-28, which have well documented roles in B-cell tumour biology and GC B-cell development^27, 28^. At 6q23.3, we observed interactions with promoter sequences upstream in *MYB* and *ALDH8A1*. At 10p14, both risk SNPs encompass a region that interacts with *TAF3*, which encodes transcription initiation factor TFIID subunit 3. TAF3 forms part of the transcription initiation factor TFIID and is necessary for haematopoiesis^29^. Finally, we observed interactions at the 16p13.13 risk locus with *RMI2* (encoding RecQ mediated genome instability 2) (Fig. 1). RMI2 is an essential component of the Bloom helicase-double Holliday junction dissolvasome and is responsible for genomic stability^30^.

**Fig. 2:**
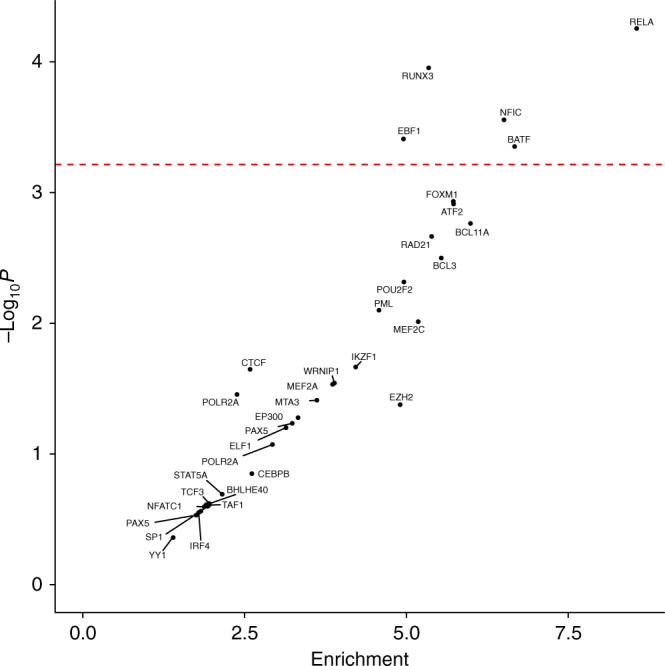
Enrichment of transcription factors binding at classical Hodgkin lymphoma risk loci. The enrichment and over-representation of transcription factors binding at all cHL risk loci. The *red line* represents the Bonferroni corrected *P*-value threshold

Across the new and established risk loci for cHL we confirmed a significant enrichment of DNase hypersensitivity in GM12878 cells (false discovery rate (FDR) adjusted *P*-value = 0.0035), as well as enhancer elements in primary B-cells (FDR adjusted *P*-value = 0.00064) and GM12878 cells (FDR adjusted *P*-value = 0.015)^31^. Analysis of ChIP-seq data on 82 transcription factors (TFs) showed an over-representation of the binding of TFs that play a central role in B-cell signalling-networks such as RELA (nuclear factor NF-kappa-B p65), EBF1 (early B-cell factor 1), RUNX3 (runt-related transcription factor 3) and BATF (basic leucine zipper transcription factor, ATF-like) (Fig. 2). Collectively, these observations support the assertion that risk loci for cHL mediate their effects through B-cell developmental networks, and are strongly involved in transcriptional initiation and enhancement.

### The HLA region

**Fig. 3:**
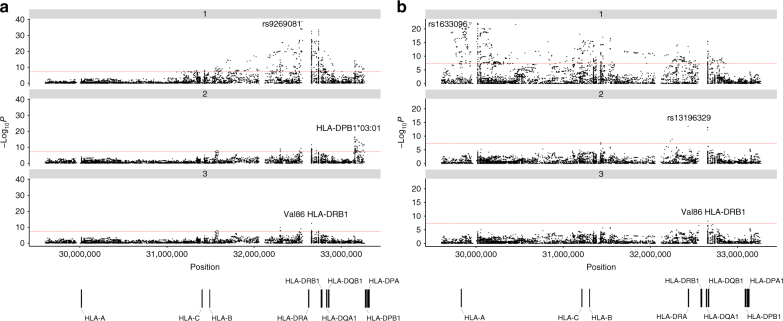
Manhattan plot representation of the step-wise conditional logistic regression of risk of **a** nodular sclerosis Hodgkin lymphoma and **b** mixed cellularity Hodgkin lymphoma within the human leukocyte antigen (HLA) region. (a1) Unconditioned test of the HLA region. (a2) Results of the HLA region after conditioning on rs9269081. (a3) Results of the HLA region after conditioning on rs9269081 and HLA-DPB1*03:01. (b1) Unconditioned test of the HLA region. (b2) Results of the HLA region after conditioning on rs1633096. (b3) Results of the HLA region after conditioning on rs1633096 and rs13196329. Physical positions are based on NCBI build 36 of the human genome. The −log_10_ of the combined logistic regression test *P*-values are plotted against their physical chromosomal position. The *broken red line* represents the genome-wide level of significance (*P* < 5 × 10^−8^)

To obtain additional insight into plausible functional variants within the major histocompatibility region at 6p21, we imputed the classical HLA alleles, amino-acid residues and SNPs using SNP2HLA^32^. To isolate independent associations for NSHL and MCHL, we performed conditional step-wise logistic regression on the strongest associated SNP, amino acid or allele, until no further variants attained genome-wide significance (Fig. 3; Supplementary Table 12). For NSHL, we identified the strongest association at rs9269081 (*P* = 1.74 × 10^−39^), which maps within the class II HLA region, 3’of HLA-DRA. Additional class II associations were shown by HLA-DPB1*03:01 (*P* = 3.35 × 10^−17^) and Val86 in HLA-DRB1 (*P* = 3.52 × 10^−13^) (Fig. 3). In contrast, the strongest association for MCHL was provided by rs1633096, a class I HLA association 3’ of HLA-F (*P* = 2.72 × 10^−23^). Additional class II associations for MCHL were observed at rs13196329, located intronic of C6orf10 (*P* = 2.58 × 10^−14^) and Val86 in HLA-DRB1 (*P* = 7.10 × 10^−9^) (Fig. 3).

### Heritability of cHL

By fitting all SNPs from GWAS simultaneously using Genome-wide Complex Trait Analysis^33^, the estimated heritability of cHL, NSHL and MCHL attributable to all common variation is 24.0% (±2.3%), 25.2% (±3.4%) and 21.9% (±2.4%), respectively. This estimate represents the additive variance, and therefore does not include the potential impact of dominance effects or gene–environment interactions having an impact on cHL risk. The currently identified non-HLA risk SNPs thus only account for around 12% of the additive heritable risk.

### Co-heritability with autoimmune disease

Although not universal, some epidemiological studies have reported associations between cHL and various autoimmune diseases, raising the possibility of common genetic susceptibility and hence common biological pathways^34^. Variation at a number of the cHL risk loci, including 3p24.1, 5q31.1 and 6q23.3 has previously been implicated as determinants of autoimmune disease risk supporting such an assertion (Supplementary Data 1).

To investigate co-heritability globally between cHL and autoimmune disease, we implemented cross-trait LD score regression^35^. Using summary-level GWAS data we estimated genetic correlations between cHL and six autoimmune diseases curated by ImmunoBase; specifically rheumatoid arthritis^36^, systemic lupus erythematosus^37^, multiple sclerosis (MS)^38^, primary biliary cirrhosis^39^, ulcerative colitis (UC)^40^ and coeliac disease^41^ GWAS data (Supplementary Table 13). We observed a positive genetic correlation between cHL and MS (*r*_g_ = 0.35, *P* = 0.04) and a negative correlation between cHL and UC (*r*_g_ = −0.23, *P* = 0.01).

## Discussion

To our knowledge, we have performed the largest GWAS of cHL to date, identifying six new non-HLA risk loci. The availability of comprehensive reference panels for the HLA region has allowed us to delineate class I and class II associations for NSHL and MCHL, substantiating recent documented differences between these cHL histologies^9^.

Although functional analyses are required to determine the biological basis of cHL association signals, we have demonstrated that these risk loci are enriched for regulatory elements in B-cells. Moreover, they feature an over-representation of key B-cell TF binding, notably RELA, RUNX3, EBF1 and BATF. RELA is a TF involved in NF-κB heterodimer formation. HRS cells show high constitutive activity of NF-κB (both canonical and non-canonical pathways)^42^, which promotes cell survival and growth through inducing anti-apoptotic and pro-proliferative gene programs^43, 44^. Inhibition of NF-κB in HRS cells leads to caspase-independent apoptosis^43^. EBF1 cooperates with E2A and PAX5 to regulate B-cell maturation^45^. Its expression in HRS cells is low^46^, which is thought to contribute to the loss of normal B-cell phenotype^47^. *RUNX3* has important roles in B-cell maturation^48^ and downregulation of *RUNX1* by *RUNX3* is required for EBV-driven LCL growth^49^. *BATF* also appears to co-ordinate B-cell maturation^50^, and is highly expressed in HRS cells^51^.

The strong HLA associations we identified for NSHL and MCHL support recent observations for distinct class I and class II relationships for these cHL subtypes^9^. Specifically, the class II NSHL association marked by rs9269081 is in strong LD with the previously identified risk SNP rs6903608 (*r*^2^ = 0.92, D’ = 1.0) for EBV-negative NSHL^9^. For MCHL the class I association rs1633096 shows correlation with the previously identified marker SNP rs2734986 (pairwise *r*^2^ = 0.41, D’ = 0.97) for EBV-positive cHL^9^. A class I association for MCHL is consistent with a high EBV positivity and supports the notion of defective cytotoxic T-cell lymphocyte responses in EBV-infected HRS cells^52^. Variation within the class II HLA region alters the risk of autoimmune diseases^53^, but the underlying biological mechanism of these associations has yet to be fully defined. The class II HLA association for NSHL and MCHL risk, comprising both coding variants and non-coding SNPs, may explain the importance of CD4+ T follicular helper (T_FH_) cells in cHL pathogenesis. In the GC, there is a requirement for CD4+ T_FH_ cells to interact with GC B-cells through the T-cell antigen receptor (TCR) and HLA class II proteins for normal plasma and memory cells formation^54^. It is therefore plausible that variation in peptide binding and expression of the HLA class II proteins contributes to cHL pathogenesis through interaction with CD4+ T_FH_ cells. Such a model is supported by the observation of variation at position 86 of HLA-DRB1 influencing TCR Vα gene expression^55^, the predominance of CD4+ T-cells in cHL tumours^56^, the reliance of HRS cells on the micro-environment for survival^1^, and the loss of MHC class II expression on HRS cells^57^, the last of which is associated with adverse prognosis. An alternative explanation for the class II HLA association in cHL is the involvement of an unidentified pathogen playing a causative role in cHL. Amino-acid variants and SNPs within HLA-DRB1 modulate humoral immune responses to common viruses, such as influenza A and JC polyomavirus^58^. Consistent with such a model is dimorphic variation at position 86 of HLA-DRB1^59^, which we identify as influencing risk of NSHL and MCHL, modulating the anchoring pocket of the antigen binding site, and influencing the conformation of peptide–DR protein complexes while maintaining a T-cell response^60^.

In our analysis we noted a reciprocal relationship between NSHL risk and ALL risk at 10p14 (*GATA3*)^22^. Since GATA3 plays a key role in B-cell development and both ALL and NSHL are malignancies derived from B-cells at different stages of maturation, our observation leads to speculation of a significant temporal effect of genetic variation at this locus in response to an environmental or mutational insult.

Although supported by a contemporaneous study and requiring further validation^61^, we found evidence for common genetic susceptibility between cHL and MS, thus raising the possibility of shared environmental risk factors. A potential biological basis for such a relationship may encompass aberrant immune activation and cell proliferation.

In conclusion, our study provides further evidence for inherited susceptibility to cHL and supports a model whereby risk loci influence disease through effects on B-cell regulatory networks, providing a mechanistic link between susceptibility and biology. Our findings also emphasise the differences between the major subtypes, which are reflective of differences in tumour aetiology.

## Methods

### Ethics

Collection of patient samples and associated clinico-pathological information was undertaken with written informed consent. Relevant ethical review boards approved the individual studies in accordance with the tenets of the Declaration of Helsinki (UK-GWAS MREC 03/1/096, German-GWAS University of Heidelberg 104/2004 and UK-GWAS-NSHLG MREC 09/MRE00/72). The diagnosis of cHL (i.e., excluding cases with nodular lymphocyte predominant HL), NSHL and MCHL (ICD-10-CM C81.1-3) in all cases was established in accordance with World Health Organisation guidelines.

### Genome-wide association studies

Primary study: We analysed constitutional DNA from 1,717 cases ascertained through the NSHLG (http://www.public.ukcrn.org.uk) from 2010 to 2013. These are detailed in Supplementary Table 1. Cases were genotyped using the Illumina Oncoarray (Illumina Inc.). Controls which were also genotyped using the oncoarray comprised: (1) 2,976 cancer-free men recruited by the PRACTICAL Consortium**—**the UK Genetic Prostate Cancer Study (UKGPCS) (age < 65 years), a study conducted through the Royal Marsden NHS Foundation Trust and SEARCH (Study of Epidemiology & Risk Factors in Cancer), recruited via GP practices in East Anglia (2003–2009), (2) 4,446 cancer-free women from across the UK via the Breast Cancer Association Consortium (BCAC).

Published studies: We used GWAS data generated on two non-overlapping case–control series of Northern European ancestry, which have been the subject of previous analyses that are summarised in Supplementary Table 1. Briefly: (1) The UK-GWAS was based on 622 cases ascertained through the Royal Marsden Hospital National Health Service Trust Family History study during 2004–2008^7^, and 5,677 controls from the UK Wellcome Trust Case Control Consortium 2 (WTCCC2)^62^. (2) The German-GWAS comprised 1,001 cases ascertained by the German Hodgkin Study Group during 1998–2007^8^, and 1,226 controls from the Heinz Nixdorf Recall (HNR) study.

### GWAS and meta-analysis

Standard quality control measures were applied to each of the three GWAS (Supplementary Tables 2, 3 and 4)^7, 8, 63^. Specifically, individuals with a low call rate (< 95%) as well as all individuals evaluated to be of non-European ancestry (using the HapMap version III CEU, JPT/CHB and YRI populations as a reference, Supplementary Fig. 1) were excluded. For apparent first-degree relative pairs, we excluded the control from a case–control pair or the individual with the lower call rate. SNPs with a call rate < 95% were excluded as were those with a MAF < 0.01 or displaying deviation from Hardy–Weinberg equilibrium (HWE) (i.e., *P* < 10^−6^, Supplementary Table 4). GWAS data were imputed to >10 million SNP with IMPUTE2 v2.3^64^ software, using a merged reference panel consisting of data from 1000 Genomes Project (phase 1 integrated release 3, March 2012)^11^ and UK10K (ALSPAC, EGAS00001000090/EGAD00001000195 and TwinsUK EGAS00001000108/EGAS00001000194 studies)^12^. HLA imputation was conducted using SNP2HLA and the Type I Diabetes Genetics Consortium reference panel of 5,225 individuals of European descent^32^. The number of variants in the HLA imputation recovered with an information measure of > 0.80 were 8,436 (94% of total variants), 8506 (95% of total variants) and 8599 (96% of total variants) in the UK-GWAS, German-GWAS and UK-NSHLG-GWAS data sets, respectively. Imputation was conducted separately for each study, and in each, the data were pruned to a common set of SNPs between cases and controls prior to imputation. Poorly imputed SNPs defined by an information measure <0.80 were excluded. Tests of association between SNPs and cHL were performed using logistic regression under an additive genetic model in SNPTESTv2.5^65^. The adequacy of the case–control matching was evaluated using Q–Q plots of test statistics (Supplementary Fig. 2). The inflation factor *λ* was based on the 90% least-significant SNP^66^. Where appropriate, principal components, generated using common SNPs, were included in the analysis to limit the effects of cryptic population stratification that otherwise might cause inflation of test statistics. Eigenvectors for the GWAS data sets were inferred using smartpca (part of EIGENSOFT) by merging cases and controls with Phase III HapMap samples. LD metrics were calculated in vcftools v0.1.12b^67^, using UK10K merged 1000 Genomes Project data and plotted using visPIG^68^.

### Replication studies and technical validation

The eight SNPs in the most promising loci (Table 1; Supplementary Table 6**)**, were taken forward for de novo replication in an additional 1,284 cases from the NSHLG and 2,504 controls from the UK replication 1 series (Supplementary Table 1**)**. After this six SNPs were genotyped in an additional replication series, (UK replication 2 series) comprising 953 cases and 565 controls from the Scotland and Newcastle Epidemiological Study of Hodgkin Disease (SNEHD), the Young Adult Hodgkin Case–Control Study (YHCCS) and the Epidemiology and Cancer Statistics Group Lymphoma Case–Control Study (ELCCS; http://www.elccs.info) (Supplementary Table 1**)**. Full details of the SNEHD, YHCCS and ELCCS studies have been previously reported^69–71^. Briefly, SNEHD involved ascertainment of incident cases from Scotland and Northern England during 1993–1997. YHCCS was based on newly diagnosed cases aged 16–24 years from Northern England during 1991–1995. ELCCS comprised cases residing in the north or parts of southwest of England aged 16–69 years with newly diagnosed, non-human immunodeficiency virus-related cHL during 1998–2003. UK population controls matched to cases on age, sex and area of residence were obtained from SNEHD, YHCCS and ELCCS. The EBV status of cHL tumours in the UK replication 2 series was determined by immunohistochemical staining for EBV latent membrane antigen-1 and/or EBV EBV-encoded RNA in situ hybridisation using sections of paraffin-embedded material^72, 73^.

The fidelity of GWAS imputation was assessed by the concordance between imputed and directly genotyped SNP in a subset of samples (Supplementary Table 7). Replication genotyping of UK samples was performed using competitive allele-specific PCR KASP chemistry (LGC, Hertfordshire, UK). Primers, probes and conditions are detailed in Supplementary Table 14. Call rates for SNP genotypes were > 95% in each of the replication series. To ensure the quality of genotyping in assays, at least two negative controls and a set of duplicates were genotyped (concordance > 99%).

### Meta-analysis

Meta-analyses were performed under a fixed-effects model using META v1.6^74^. Cochran’s Q-statistic to test for heterogeneity and the *I*^2^ statistic to quantify the proportion of the total variation due to heterogeneity were calculated; an *I*^2^ value ≥ 75% is considered to be characteristic of large heterogeneity^75^. We used the test-based method of Higgins et al.^76^ to derive 95% CIs for *I*^2^ values (Supplementary Table 9). To estimate study power of the discovery GWAS phase, we made use of the CaTS online calculator^77^, assuming a risk allele frequency of 0.2 and genotype relative risk of 1.20.

### Expression quantitative trait locus analysis

To examine the relationship between SNP genotype and gene expression, we carried out SMR analysis as per Zhu et al., 2016^25^. Briefly, if *b*_*xy*_ is the effect size of *x* (gene expression) on *y* (slope of *y* regressed on the genetic value of *x*), *b*_*zx*_ is the effect of *z* on *x*, and *b*_*zy*_ is the effect of *z* on *y*. Therefore *b*_*xy*_ (*b*_*zy*_/*b*_*zx*_) is the effect of *x* on *y*. To distinguish pleiotropy from linkage where the top associated *cis*-eQTL is in LD with two causal variants, one affecting gene expression the other affecting trait, we tested for heterogeneity in dependent instruments, using multiple SNPs in each *cis*-eQTL region. Under the hypothesis of pleiotropy *b*_*xy*_ values for SNPs in LD with the causal variant will be identical. Thus testing against the null hypothesis that there is a single causal variant is equivalent to testing heterogeneity in the *b*_*xy*_ values estimated for the SNPs in the *cis*-eQTL region. For each probe that passed significance threshold for the SMR test, we tested the heterogeneity in the *b*_*xy*_ values estimated for multiple SNPs in the *cis*-eQTL region using HEIDI.

We used publicly available LCL expression data from the MuTHER (*n* = 825)^23^ and GTEx consortium (*n* = 114)^24^. Briefly, GWAS summary statistics files were generated from the meta-analysis. Reference files were generated from merging 1000 Genomes Project phase 3 and UK10K (ALSPAC and TwinsUK) vcfs^11, 12^. As previously advocated, only probes with at least one eQTL *P*-value of < 5.0 × 10^−8^ were considered for SMR analysis^25^. We set a threshold for the SMR test of *P*_SMR_ < 5.49 × 10^−4^ corresponding to a Bonferroni correction for 91 tests (91 probes with a top eQTL *P* < 5.0 × 10^−8^ across the 12 loci and two LCL eQTL data sets). For all genes passing this threshold, we generated plots of the eQTL and GWAS associations at the locus, as well as plots of GWAS and eQTL effect sizes (i.e., corresponding to input for the HEIDI heterogeneity test). HEIDI test *P*-values < 0.05 were considered as being reflective of heterogeneity. This threshold is conservative for gene discovery because it retains fewer genes than when correcting for multiple testing. SMR plots for significant eQTLs are shown in Supplementary Fig. 5.

### Chromatin state dynamics

Enrichment of cHL risk SNPs with DNAse and enhancers is conducted using Haploreg v4^31^. The overlap of cHL risk SNPs with enhancers in GM12878 cell is compared to a background model of all 1000 Genomes Project variants with a frequency above 5% in any population. The enrichment relative to these background frequencies was performed using a binomial test and a FDR *P*-value was subsequently calculated; we considered an FDR < 0.05 as being significant.

To examine enrichment in specific TF binding across risk loci, we adapted the variant set enrichment method of Cowper-Sal lari et al.^78^. For each risk locus, a region of strong LD (defined as *r*^2^ > 0.8 and *D*′ > 0.8) was determined, and these SNPs were termed the associated variant set (AVS). TF ChIP-seq uniform peak data were obtained from ENCODE for the GM12878 cell line, and included data for 82 TFs. For each of these marks, the overlap of the SNP in the AVS and the binding sites was determined to produce a mapping tally. A null distribution was produced by randomly selecting SNP with the same LD structure (generated from 1000 Genomes Project and UK10K data) as the risk associated SNP, and the null mapping tally calculated. This process was repeated 10,000 times, and approximate *P*-values were calculated as the proportion of permutations where the null mapping tally was greater or equal to the AVS mapping tally. An enrichment score was calculated by the tallies to the median of the null distribution. Thus the enrichment score is the number of standard deviations of the AVS mapping tally from the mean of the null distribution tallies.

### Promoter capture Hi-C data

To map risk SNPs to interactions involving promoter contacts and identify genes involved in cHL susceptibility, we analysed promoter capture Hi-C data on the LCL cell line GM12878 as a model B-cell^26^. Reads from technical replicates (E-MTAB-2323) were combined before processing with HiCUP^79^. Significant interactions (i.e., score ≥ 5) on two biological replicates were determined using CHiCAGO^80^.

### Co-heritability of Hodgkin lymphoma with autoimmune disease

We utilised LD regression to estimate genetic correlation between individual autoimmune diseases and cHL, NSHL and MCHL^35^. Summary statistics for published studies of coeliac disease^41^, systemic lupus erythematosus^37^, primary biliary cirrhosis^39^, rheumatoid arthritis^36^, MS^38^ and UC^40^ were downloaded from the ImmunoBase website (http://www.immunobase.org/).

### Heritability analysis

We used genome-wide complex trait analysis to estimate the polygenic variance (i.e., heritability) ascribable to all genotyped and imputed GWAS SNPs^33^. SNPs were excluded based on low MAF < 0.01, poor imputation (info score < 0.9) and evidence of departure from HWE (*P* < 0.05). Individuals were excluded for poor imputation and where two individuals were closely related. A genetic relationship matrix of pairs of samples was used as input for the restricted maximum likelihood analysis to estimate the heritability explained by the selected set of SNPs. To transform the estimated heritability to the liability scale, we used the lifetime risk, for cHL, which is estimated to be 0.002 by SEER (https://seer.cancer.gov/statfacts/html/hodg.html).

### Data availability

Genotype data that support the findings of this study have been deposited in the European Genome-phenome Archive (EGA) under accession codes EGAD00000000022 and EGAD00000000024.

Sequencing data, which forms the reference panel for imputation, have been deposited in the European Genome-phenome Archive (EGA) under accession codes EGAS00001000090, EGAD00001000195, EGAS00001000108.

Transcriptional profiling data from the MuTHER consortium that support the findings of this work have been deposited in the European Bioinformatics Institute (Part of the European Molecular Biology Laboratory, EMBL-EBI) under accession code E-TABM-1140.

Transcriptional profiling data from the Genotype-Tissue Expression (GTEx) project, that support the findings of this work are available here: https://www.gtexportal.org/

Transcription factor binding data that support the findings of this work are available here: http://genome.ucsc.edu/ENCODE/downloads.html.

Promoter capture Hi-C data in GM12878 cells that support the findings of this work have been deposited in the European Bioinformatics Institute (Part of the European Molecular Biology Laboratory, EMBL-EBI) under accession code E-MTAB-2323.

The remaining data contained within the paper and supplementary files are available from the author upon request.

## Electronic supplementary material

Supplementary Information

Supplementary Data 1
