# Author Correction: Genome-wide association study of classical Hodgkin lymphoma identifies key regulators of disease susceptibility

**DOI:** 10.1038/s41467-018-08105-w

**Published:** 2019-01-08

**Authors:** Amit Sud, Hauke Thomsen, Philip J. Law, Asta Försti, Miguel Inacio da Silva Filho, Amy Holroyd, Peter Broderick, Giulia Orlando, Oleg Lenive, Lauren Wright, Rosie Cooke, Douglas Easton, Paul Pharoah, Alison Dunning, Julian Peto, Federico Canzian, Rosalind Eeles, Zsofia Kote-Jarai, Kenneth Muir, Nora Pashayan, The PRACTICAL consortium, Brian E. Henderson, Christopher A. Haiman, Sara Benlloch, Fredrick R. Schumacher, Ali Amin Al Olama, Sonja I. Berndt, David V. Conti, Fredrik Wiklund, Stephen Chanock, Victoria L. Stevens, Catherine M. Tangen, Jyotsna Batra, Judith Clements, Henrik Gronberg, Johanna Schleutker, Demetrius Albanes, Stephanie Weinstein, Alicja Wolk, Catharine West, Lorelei Mucci, Géraldine Cancel-Tassin, Stella Koutros, Karina Dalsgaard Sorensen, Lovise Maehle, David E. Neal, Ruth C. Travis, Robert J. Hamilton, Sue Ann Ingles, Barry Rosenstein, Yong-Jie Lu, Graham G. Giles, Adam S. Kibel, Ana Vega, Manolis Kogevinas, Kathryn L. Penney, Jong Y. Park, Janet L. Stanford, Cezary Cybulski, Børge G. Nordestgaard, Hermann Brenner, Christiane Maier, Jeri Kim, Esther M. John, Manuel R. Teixeira, Susan L. Neuhausen, Kim De Ruyck, Azad Razack, Lisa F. Newcomb, Davor Lessel, Radka Kaneva, Nawaid Usmani, Frank Claessens, Paul A. Townsend, Manuela Gago-Dominguez, Monique J. Roobol, Florence Menegaux, Per Hoffmann, Markus M. Nöthen, Karl-Heinz Jöckel, Elke Pogge von Strandmann, Tracy Lightfoot, Eleanor Kane, Eve Roman, Annette Lake, Dorothy Montgomery, Ruth F. Jarrett, Anthony J. Swerdlow, Andreas Engert, Nick Orr, Kari Hemminki, Richard S. Houlston

**Affiliations:** 1 Division of Genetics and Epidemiology, The Institute of Cancer Research, London, SW7 3RP UK; 2 Division of Molecular Genetic Epidemiology, German Cancer Research Centre, Heidelberg, 69120 Germany; 3 Centre for Primary Health Care Research, Lund University, Malmö, 221 00 Sweden; 4 Centre for Cancer Genetic Epidemiology, Department of Oncology, University of Cambridge, Cambridge, CB1 8RN UK; 5 Centre for Cancer Genetic Epidemiology, Department of Public Health and Primary Care, University of Cambridge, Cambridge, CB1 8RN UK; 6 Department of Non-Communicable Disease Epidemiology, London School of Hygiene and Tropical Medicine, London, WC1E 7HT UK; 7 Genomic Epidemiology Group, German Cancer Research Center (DKFZ), Heidelberg, 69120 Germany; 8 Royal Marsden NHS Foundation Trust, London, SM2 5NG UK; 9 Institute of Population Health, University of Manchester, Manchester, M1 3BB UK; 10 Division of Health Sciences, Warwick Medical School, Warwick University, Warwick, CV4 7AL UK; 11 Department of Applied Health Research, University College London, London, WC1E 7HB UK; 12 Department of Biomedicine, Division of Medical Genetics, University of Basel, Basel, 4031 Switzerland; 13 Institute of Human Genetics, University of Bonn, Bonn, 53127 Germany; 14 Department of Genomics, Life & Brain Center, University of Bonn, Bonn, 53127 Germany; 15 University of Duisburg–Essen, Essen, 47057 Germany; 16 Department of Internal Medicine, University Hospital of Cologne, Cologne, 50937 Germany; 17 Department of Health Sciences, University of York, York, YO10 5DD UK; 18 MRC University of Glasgow Centre for Virus Research, Glasgow, G61 1QH UK; 19 Division of Breast Cancer Research, The Institute of Cancer Research, London, SW7 3RP UK; 20 Division of Molecular Pathology, The Institute of Cancer Research, London, SW7 3RP UK; 21 Department of Preventive Medicine, Keck School of Medicine, University of Southern California/Norris Comprehensive Cancer Center, Los Angeles, CA 90033 USA; 22 Department of Epidemiology and Biostatistics, Case Western Reserve University, Cleveland, OH 44106 USA; 23 Seidman Cancer Center, University Hospitals, Cleveland, OH 44106 USA; 24 Department of Clinical Neurosciences, University of Cambridge, Cambridge, CB2 1TN UK; 25 Division of Cancer Epidemiology and Genetics, National Cancer Institute, NIH, Bethesda, MD 20892 USA; 26 Department of Medical Epidemiology and Biostatistics, Karolinska Institute, SE-171 77 Stockholm, Sweden; 27 Epidemiology Research Program, American Cancer Society, 250 Williams Street, Atlanta, Georgia 30303 USA; 28 SWOG Statistical Center, Fred Hutchinson Cancer Research Center, Seattle, WA 98109 USA; 29 Australian Prostate Cancer Research Centre-Qld, Institute of Health and Biomedical Innovation and School of Biomedical Science, Queensland University of Technology, Brisbane, QLD 4059 Australia; 30 Translational Research Institute, Brisbane, QLD 4102 Australia; 31 Department of Medical Biochemistry and Genetics, Institute of Biomedicine, University of Turku, FI-20520 Turku, Finland; 32 Tyks Microbiology and Genetics, Department of Medical Genetics, Turku University Hospital, FI-20520 Turku, Finland; 33 BioMediTech, University of Tampere, Tampere, 33100 Finland; 34 Division of Nutritional Epidemiology, Institute of Environmental Medicine, Karolinska Institutet,, SE-171 77 Stockholm, Sweden; 35 Institute of Cancer Sciences, University of Manchester, Manchester Academic Health Science Centre, Radiotherapy Related Research, The Christie Hospital NHS Foundation Trust, Manchester, M13 9NT UK; 36 Department of Epidemiology, Harvard School of Public Health, Boston, MA 02115 USA; 37 CeRePP, Pitie-Salpetriere Hospital, 75013 Paris, France; 38 UPMC Univ Paris 06, GRC N°5 ONCOTYPE-URO, CeRePP, Tenon Hospital, 75020 Paris, France; 39 Department of Molecular Medicine, Aarhus University Hospital, 8000 Aarhus C, Denmark; 40 Department of Clinical Medicine, Aarhus University, 8000 Aarhus C, Denmark; 41 Department of Medical Genetics, Oslo University Hospital, N-0424 Oslo, Norway; 42 Department of Oncology, Addenbrooke’s Hospital, University of Cambridge, Cambridge, CB2 0QQ UK; 43 Cancer Research UK, Cambridge Research Institute, Li Ka Shing Centre, Cambridge, CB2 0RE UK; 44 Cancer Epidemiology, Nuffield Department of Population Health, University of Oxford, Oxford, OX3 7LF UK; 45 Dept. of Surgical Oncology, Princess Margaret Cancer Centre, Toronto, ON M5G 2M9 Canada; 46 Department of Radiation Oncology, Icahn School of Medicine at Mount Sinai, New York, NY 10029 USA; 47 Department of Genetics and Genomic Sciences, Icahn School of Medicine at Mount Sinai, New York, NY 10029 USA; 48 Centre for Molecular Oncology, Barts Cancer Institute, Queen Mary University of London, John Vane Science Centre, London, EC1M 6BQ UK; 49 Cancer Epidemiology Centre, The Cancer Council Victoria, Melbourne, VIC 3004 Australia; 50 Centre for Epidemiology and Biostatistics, Melbourne School of Population and Global Health, The University of Melbourne, Melbourne, VIC 3053 Australia; 51 Division of Urologic Surgery, Brigham and Womens Hospital, Boston, MA 02115 USA; 52 Fundación Pública Galega de Medicina Xenómica-SERGAS, Grupo de Medicina Xenómica, CIBERER, IDIS, 15782 Santiago de Compostela, Spain; 53 Centre for Research in Environmental Epidemiology (CREAL), Barcelona Institute for Global Health (ISGlobal), 60803 Barcelona, Spain; 54 CIBER Epidemiología y Salud Pública (CIBERESP), 28029 Madrid, Spain; 55 IMIM (Hospital del Mar Research Institute), 08003 Barcelona, Spain; 56 Universitat Pompeu Fabra (UPF), 08002 Barcelona, Spain; 57 Channing Division of Network Medicine, Department of Medicine, Brigham and Women’s Hospital/Harvard Medical School, Boston, MA 02115 USA; 58 Department of Cancer Epidemiology, Moffitt Cancer Center, Tampa, FL 33612 USA; 59 Division of Public Health Sciences, Fred Hutchinson Cancer Research Center, Seattle, WA 98109 USA; 60 Department of Epidemiology, School of Public Health, University of Washington, Seattle, WA 98195 USA; 61 International Hereditary Cancer Center, Department of Genetics and Pathology, Pomeranian Medical University, 70-001 Szczecin, Poland; 62 Faculty of Health and Medical Sciences, University of Copenhagen, 1165 Copenhagen, Denmark; 63 Department of Clinical Biochemistry, Herlev and Gentofte Hospital, University Hospital, 2900 Copenhagen, Denmark; 64 Division of Clinical Epidemiology and Aging Research, German Cancer Research Center (DKFZ), 69120 Heidelberg, Germany; 65 German Cancer Consortium (DKTK), German Cancer Research Center (DKFZ), 69120 Heidelberg, Germany; 66 Division of Preventive Oncology, German Cancer Research Center (DKFZ) and National Center for Tumor Diseases (NCT), 69120 Heidelberg, Germany; 67 Institute for Human Genetics, University Hospital Ulm, 89081 Ulm, Germany; 68 Department of Genitourinary Medical Oncology, The University of Texas M. D. Anderson Cancer Center, Houston, TX 77030 USA; 69 Cancer Prevention Institute of California, Fremont, CA 94538 USA; 70 Department of Health Research & Policy (Epidemiology) and Stanford Cancer Institute, Stanford University School of Medicine, Stanford, CA 94305 USA; 71 Department of Genetics, Portuguese Oncology Institute of Porto, 4200-072 Porto, Portugal; 72 Biomedical Sciences Institute (ICBAS), University of Porto, 4200-072 Porto, Portugal; 73 Department of Population Sciences, Beckman Research Institute of the City of Hope, Duarte, CA 91016 USA; 74 Faculty of Medicine and Health Sciences, Basic Medical Sciences, Ghent University, 9000 Ghent, Belgium; 75 Department of Surgery, Faculty of Medicine, University of Malaya, 50603 Kuala Lumpur, Malaysia; 76 Department of Urology, University of Washington, Seattle, WA 98105 USA; 77 Institute of Human Genetics, University Medical Center Hamburg-Eppendorf, 20246 Hamburg, Germany; 78 Molecular Medicine Center, Department of Medical Chemistry and Biochemistry, Medical University, 1431 Sofia, Bulgaria; 79 Department of Oncology, Cross Cancer Institute, University of Alberta, Edmonton, AB T6G 2R3 Canada; 80 Division of Radiation Oncology, Cross Cancer Institute, Edmonton, AB T6G 1Z2 Canada; 81 Molecular Endocrinology Laboratory, Department of Cellular and Molecular Medicine, KU Leuven, 3000 Leuven, Belgium; 82 Institute of Cancer Sciences, Manchester Cancer Research Centre, University of Manchester, Manchester Academic Health Science Centre, St Mary’s Hospital, Manchester, M13 9WL UK; 83 Genomic Medicine Group, Galician Foundation of Genomic Medicine, Instituto de Investigacion Sanitaria de Santiago de Compostela (IDIS), Complejo Hospitalario Universitario de Santiago, Servicio Galego de Saúde, SERGAS, 15706 Santiago De Compostela, Spain; 84 University of California San Diego, Moores Cancer Center, La Jolla, CA 92093 USA; 85 Department of Urology, Erasmus University Medical Center, 3015 CE Rotterdam, The Netherlands; 86 Cancer & Environment Group, Center for Research in Epidemiology and Population Health (CESP), INSERM, University Paris-Sud, University Paris-Saclay, F-94805 Villejuif, France

Correction to: *Nature Communications*; 10.1038/s41467-017-00320-1; published online 1 December 2017

The original version of this Article contained an error in the spelling of a member of the PRACTICAL Consortium, Manuela Gago-Dominguez, which was incorrectly given as Manuela Gago Dominguez. This has now been corrected in both the PDF and HTML versions of the Article.

