# Supplementary material for: Genome-wide association study of classical Hodgkin lymphoma identifies key regulators of disease susceptibility
Source: Nat Commun. 2017 Dec 1;8:1892. doi: 10.1038/s41467-017-00320-1 (PMC5711884; doi:10.1038/s41467-017-00320-1)
Supplement: Supplementary file 1 — Supplementary Information [file 41467_2017_320_MOESM1_ESM.pdf]

File name: Supplementary Information
Description: Supplementary Figures, Supplementary Tables and Supplementary References

File name: Supplementary Data 1
Description: Associations of autoimmune disease and B-cell malignancy non-HLA risk SNP at classical Hodgkin lymphoma genomic risk loci

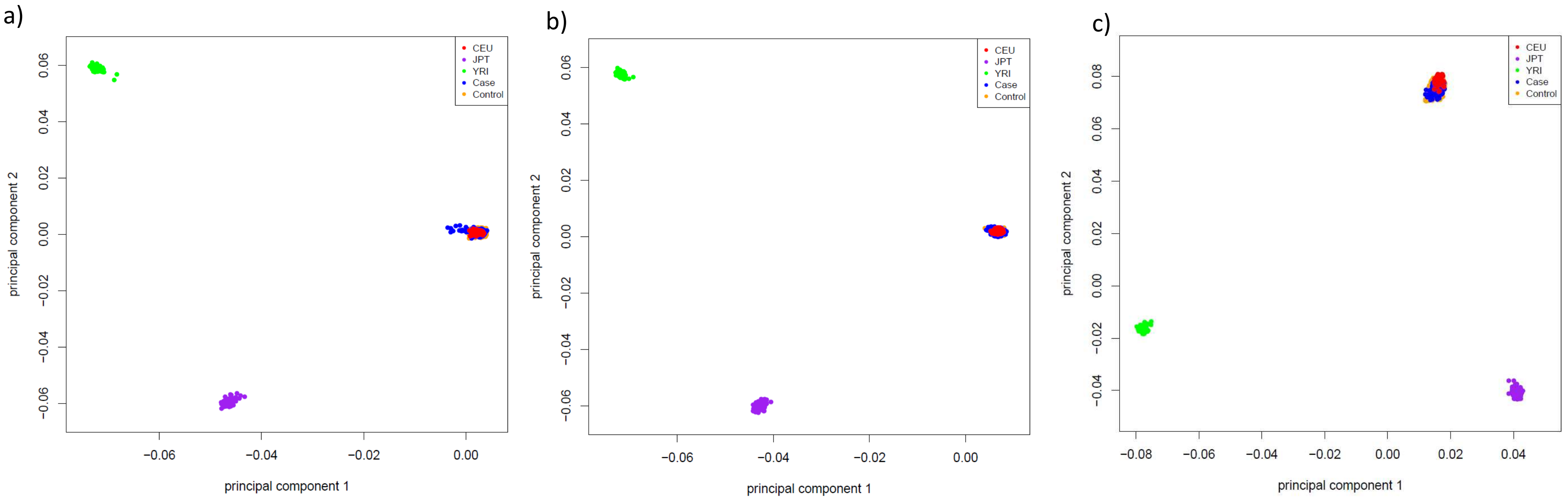

Supplementary Figure 1: Identification of individuals of non-European ancestry in cases and controls. (a) UK-GWAS (b) German-GWAS (c) UK-NSHLG-GWAS. The first two principal components of the analysis are plotted. HapMap CEU individuals are plotted in red, JPT individuals are plotted in indigo, YRI are plotted in green. Cases are plotted in blue, controls plotted in orange.

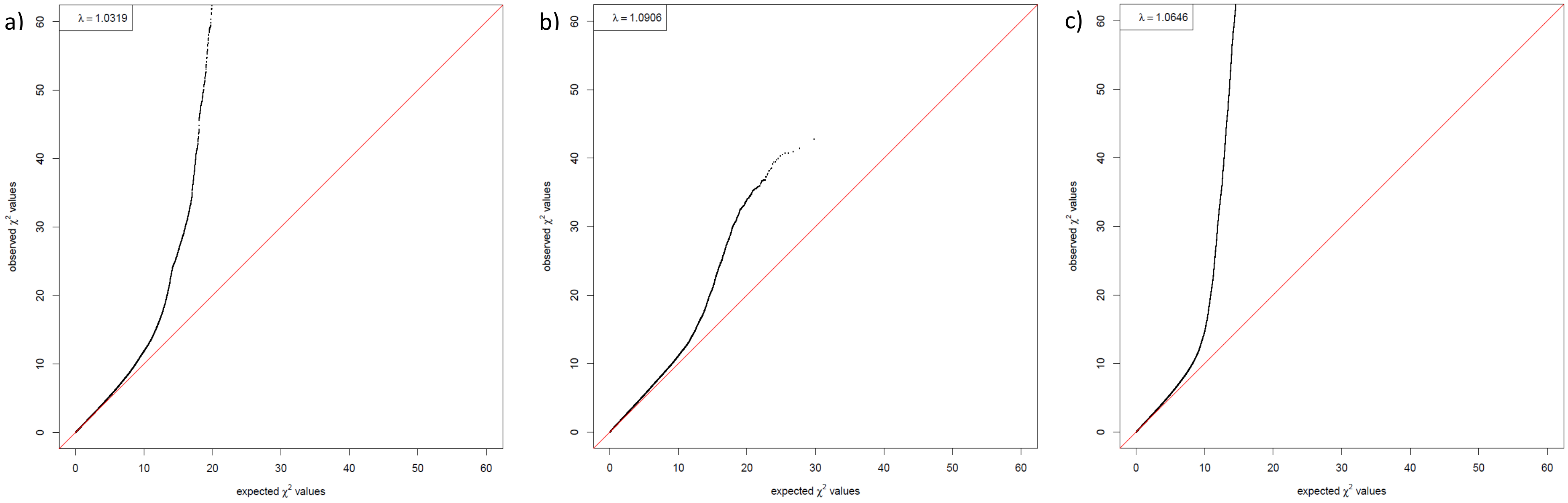

Supplementary Figure 2: Quantile-Quantile plots of observed and expected χ^2 values of association between SNP genotype and risk of classical Hodgkin lymphoma after imputation. a) UK-GWAS, b) German-GWAS, c) UK-NSHLG-GWAS. The red line represents the null hypothesis of no true association.

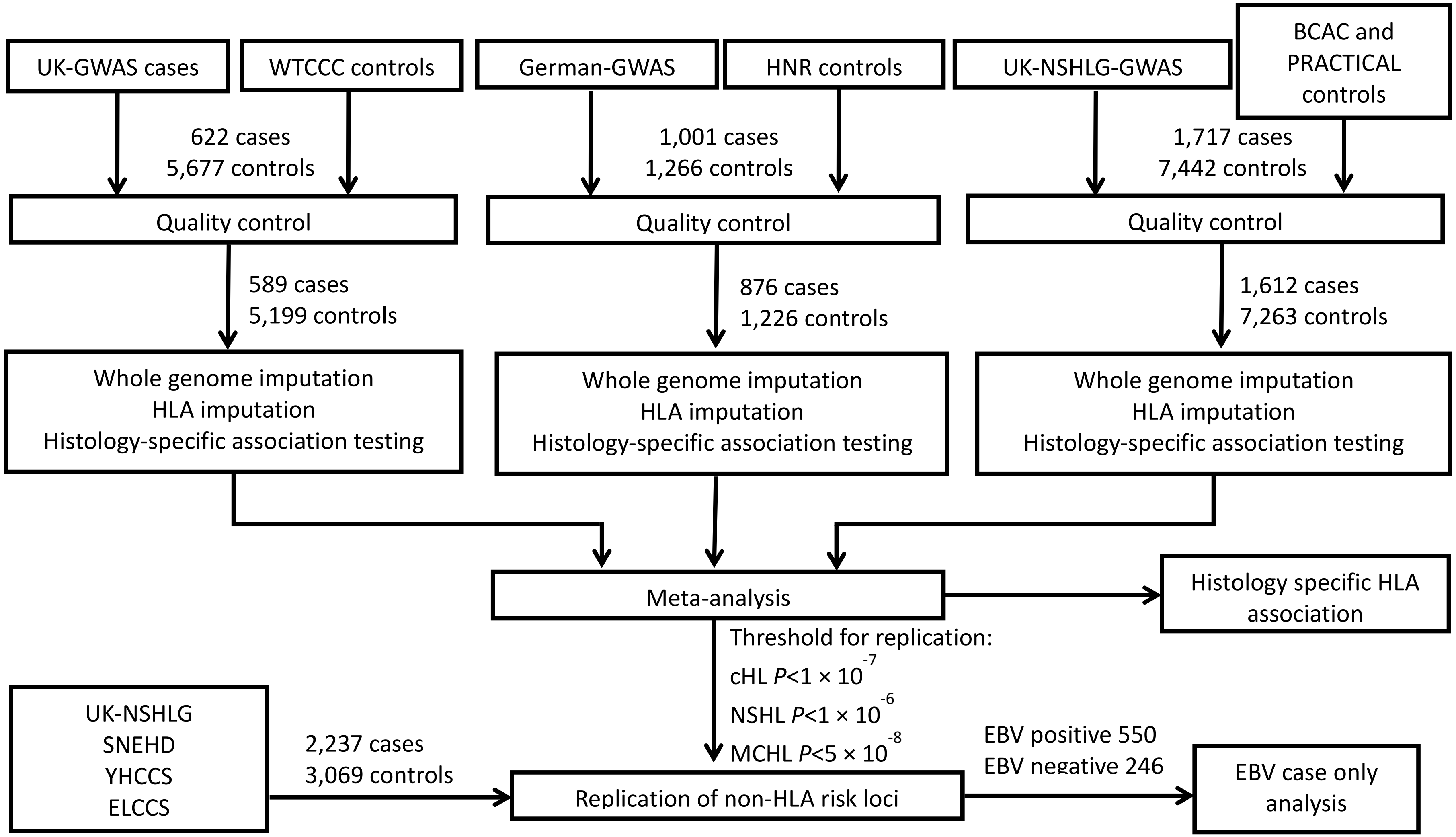

Supplementary Figure 3: Analysis strategy for classical Hodgkin lymphoma genome-wide association study. WTCCC, Wellcome Trust Case Control Consortium; HNR, Heinz-Nixdorf Recall; NSHLG, National Study of Hodgkin lymphoma Genetics; BCAC The Breast Cancer Association Consortium; PRACTICAL, Prostate Cancer Association Group to Investigate Cancer Associated Alterations in the Genome; SNEHD, Scotland and Newcastle Epidemiological Study of Hodgkin Disease; YHCCS, Young Adult Hodgkin Case-Control Study; ELCCS, Epidemiology and Cancer Statistics Group Lymphoma Case-Control Study; cHL, classical Hodgkin lymphoma; NSHL, nodular sclerosis Hodgkin lymphoma; MCHL, mixed cellularity Hodgkin lymphoma; HLA, human leucocyte antigen; EBV, Epstein-Barr virus.

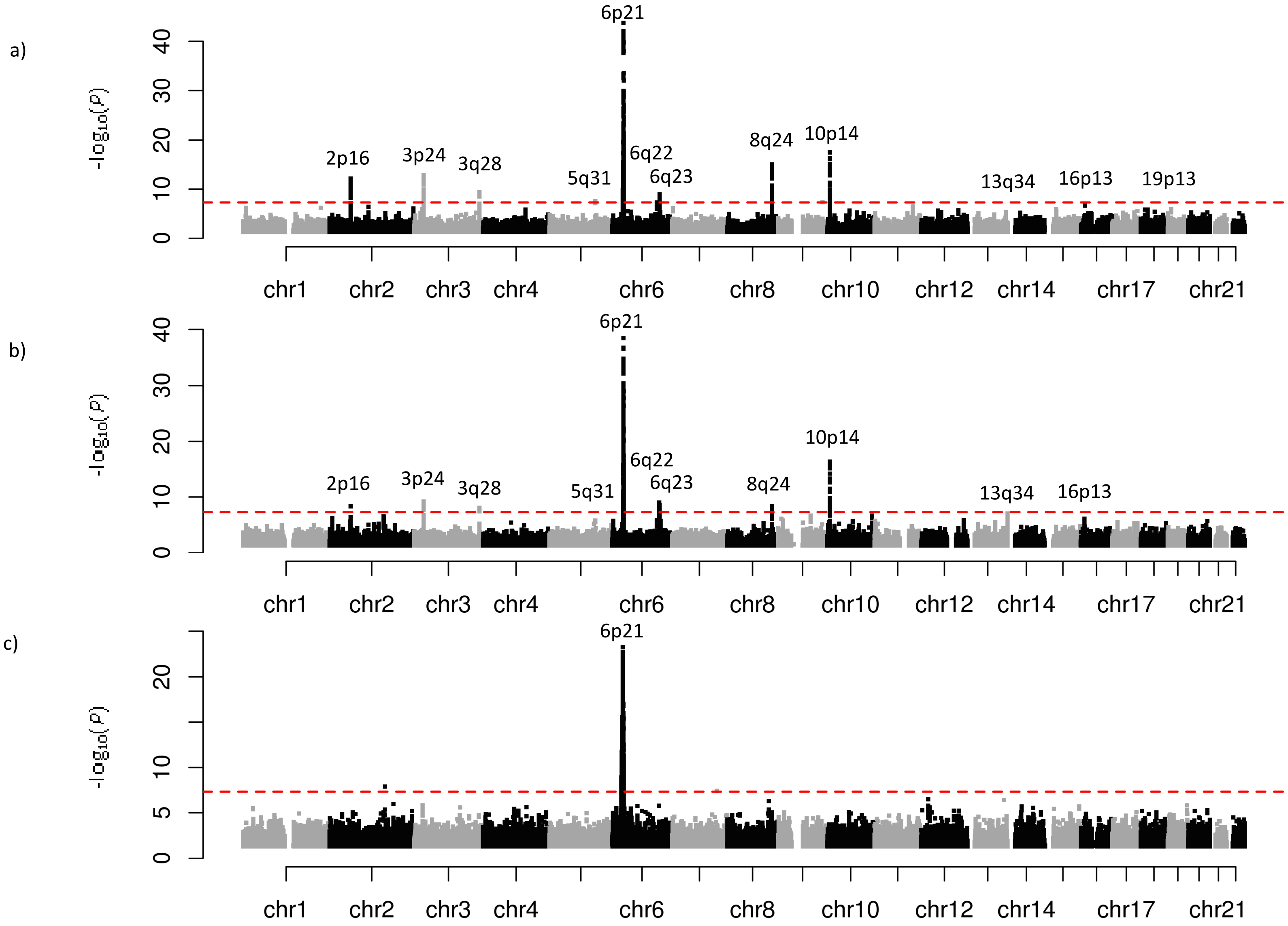

Supplementary Figure 4: Manhattan plot of association *P*-values for a) classical Hodgkin lymphoma, b) nodular sclerosis Hodgkin lymphoma c) mixed cellularity Hodgkin lymphoma. Shown are the genome-wide *P*-values (two sided) of imputed autosomal SNPs from the respective discovery-phase GWAS. Labelled are identified risk loci. The red horizontal line represents the genome-wide significance threshold of $P=5 \times 10^{-8}$.

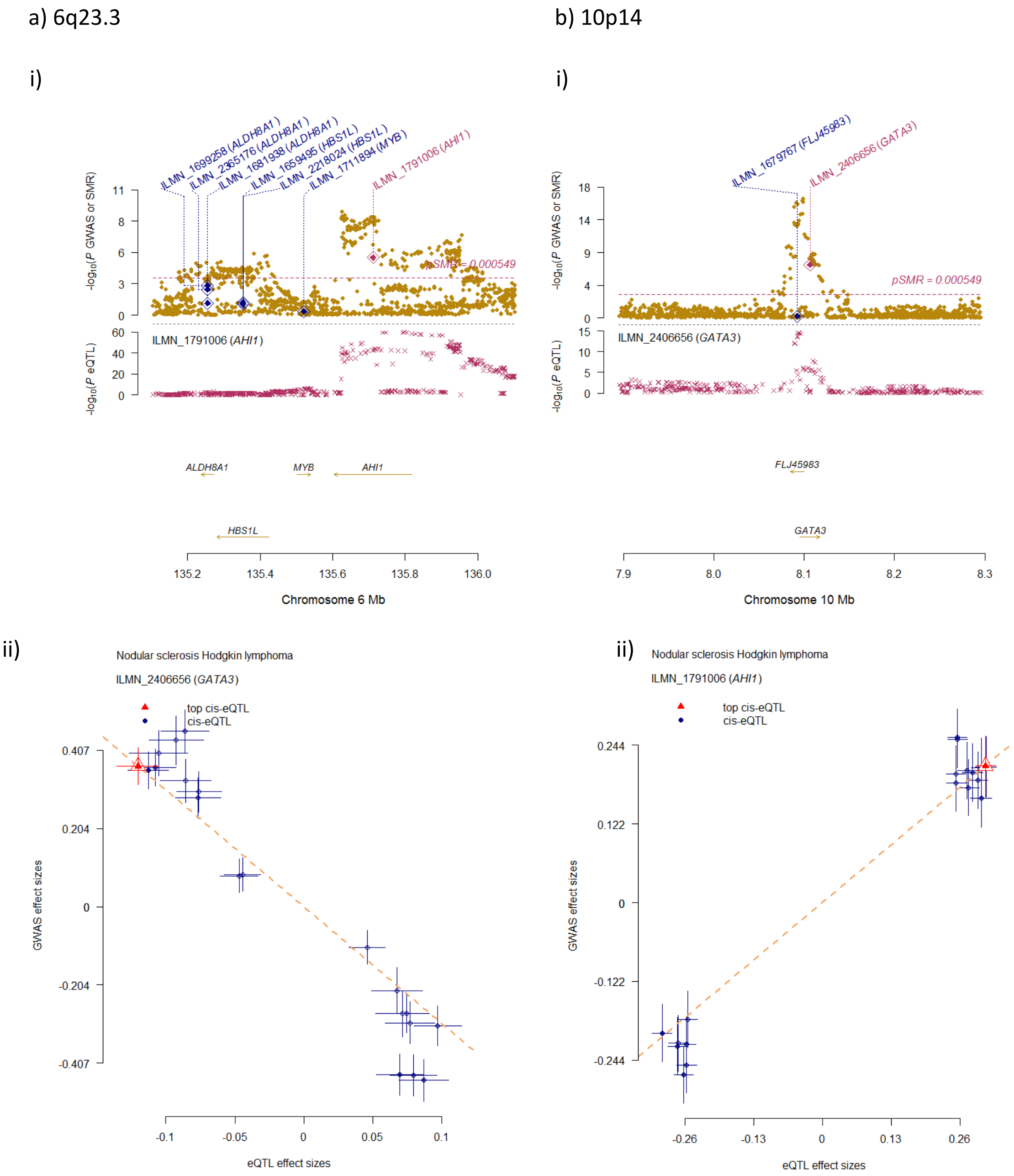

Supplementary Figure 5: Summary-data-based Mendelian randomization analysis of classical Hodgkin lymphoma risk loci and gene expression in lymphoblastoid cell lines a) 6q23.3 b) 10p14. Upper panel - brown dots represent *P*-values for single nucleotide polymorphisms (SNPs) from the meta-analysis, diamonds represent *P*-values for probes from the summary-data-based Mendelian randomization (SMR) test. Lower panel - crosses represent expression quantitative trait loci (eQTL) *P*-values of SNPs from respective tissue, genes passing the SMR (i.e. $P_{SMR}< 5.49 \times 10^{-4}$) and HEIDI (i.e. $P_{HEIDI}>0.05$) tests are highlighted in red, the top and bottom plots include all SNPS mapping to the region in the GWAS and eQTL summary data, rather than only the SNPs common to both data sets (ii) Effect sizes of SNPs (used for the HEIDI test) from GWAS meta-analysis plotted against those for SNPs from the respective eQTL study. The orange dashed lines correspond to the estimate of b_{xy}, at the top *cis*-eQTL. Error bars correspond to standard errors of SNP effects.

Study	Study centre	Country of enrolment	Cases	Controls
Discovery UK-GWAS[1]	The Institute of Cancer Research	UK	622 cHL cases (63 male, mean age at diagnosis = 24.4 years, 189 NSHL cases) from the Royal Marsden Hospitals National Health Service Trust Family History/ ICR study (2004–2008)<br>EBV status NA	2,930 from the 1958 Birth Cohort<br>2,737 from the National Blood Service
Discovery German-GWAS[2]	The German Cancer Research Centre<br>University of Cologne	Germany	1,001 cHL cases (597 male, mean age at diagnosis = 34.8 years, 417 NSHL cases, 180 MCHL cases, 155 young adult NSHL) from the German Hodgkin study group<br>EBV status NA	1,226 from the Heinz-Nixdorf Recall Study
Discovery UK-NSHLG-GWAS	The Institute of Cancer Research	UK	1,717 cHL cases (754 male, mean age at diagnosis = 41.5 years, 720 NSHL cases, 637 MCHL cases, 270 young adult NSHL) from the NSHLG (2010-2013)<br>EBV status NA	2,976 from the Prostate Cancer Association Group to Investigate Cancer Associated Alterations in the Genome consortium<br>4,446 from The Breast Cancer Association Consortium consortium.
Replication 1	The Institute of Cancer Research	UK	1,284 cHL cases (655 male, mean age at diagnosis = 41.2 years, 1,258 NSHL cases, 561 young adult NSHL cases) from the NSHLG (2010-2013)<br>EBV status NA	2,504 from the National Study of Colorectal Cancer Genetics and Genetic Lung Cancer Predisposition Study.[3,4]
Replication 2[5,6]	Medical Research Council University of Glasgow Centre for Virus Research<br>Department of Health Sciences, University of York	UK	953 cHL cases (309 male, mean age at diagnosis = 38.0 years, 329 NSHL cases, 109 MCHL cases, 118 young adult NSHL cases, 550 EBV positive cases, 246 EBV negative cases) from the Scotland and Newcastle Epidemiological Study of Hodgkin Disease, the Young Adult Hodgkin Case–Control Study and the Epidemiology and Cancer Statistics Group Lymphoma Case–Control Study	565 UK population controls matched by age, sex and area of residence.

Supplementary Table 1: Details of each case-control series. Young adult nodular sclerosis Hodgkin lymphoma < 35 years of age at diagnosis. NSHLG, National Study of Hodgkin Lymphoma Genetics; cHL, classical Hodgkin lymphoma; NSHL, nodular sclerosis Hodgkin lymphoma; MCHL, mixed cellularity Hodgkin lymphoma; EBV, Epstein-Barr virus; NA, not available.

	UK-GWAS		German-GWAS		UK-NSHLG-GWAS	
	Cases	Controls	Cases	Controls	Cases	Controls
Pre-quality control	622	5,677	1,001	1,226	1,717	7,422
Sex discrepancy	3	25	11	2	14	0
Call rate fail	12	248	9	0	3	12
Heterozygosity rate	NA	NA	27	2	25	43
Other*	NA	65	NA	NA	NA	NA
Related Individuals	2	71	19	2	14	66
None-European Ancestry	30	108	86	2	59	135
Post-quality control†	589	5,199	876	1,218	1,612	7,263

Supplementary Table 2: Details of the quality control filters applied to each genome-wide association study. Samples were excluded due to call rate (< 95% or failed genotyping), ethnicity (principle components analysis or other samples reported to be not of white, European descent), relatedness (any individuals found to be duplicated or related within or between data sets through identity by state) or sex discrepancy. NSHLG, National Study of Hodgkin lymphoma Genetics.

* channel problem and unknown identity (see www.wtccc.org.uk for details), 1 individual in 58BC diagnosed with cHL

† filters for quality control were performed simultaneously so numbers for each criteria may not sum to total removed.

	UK-GWAS	German-GWAS	UK-NSHLG-GWAS	UK replication 1	UK replication 2
Classical Hodgkin lymphoma	589	876	1,612	1284	953
Nodular sclerosis Hodgkin lymphoma	155 (26%)	402 (46%)	721 (45%)	1258 (98%)	328 (34%)
Mixed cellularity Hodgkin lymphoma	12 (2%)	179 (20%)	637 (40%)	26 (2%)	109 (11%)
Lymphocyte-rich Hodgkin lymphoma	5 (1%)	0 (0%)	7 (<1%)	0 (0%)	3 (<1%)
Lymphocyte depleted Hodgkin lymphoma	8 (1%)	0 (0%)	0 (0%)	0 (0%)	0 (0%)
Unspecified	409 (71%)	295 (34%)	247 (15%)	0 (0%)	513 (54%)
Epstein-Barr virus negative	NA	NA	NA	NA	550 (58%)
Epstein-Barr virus positive	NA	NA	NA	NA	246 (26%)

Supplementary Table 3: Histology of the cases in each genome-wide association study and replication following quality control. GWAS, genome-wide association study; NSHLG, National Study of Hodgkin lymphoma Genetics; NA, not available.

	UK-GWAS			German-GWAS	UK-NSHLG-GWAS
	Cases	Controls			
		1958BC	UKBS		
Genotyping Platform	Illumina 660W-Quad BeadChips	Illumina Human1.2M-Duo Custom_v1 Array BeadChips		Illumina Human OmniExpress-12 v1.0	Infinium OncoArray-500K BeadChip
Pre-quality control	640,663	1,115,428	1,115,428	733,202	464,624
Call rate fail	99,296	18,418	20,224	2,083	11
HWE fail	867	11,662	14,374	1,104	14
MAF < 0.01	24,177	179,500	178,713	78,364	73,351
Other*	n/a	189,910	183,751	n/a	n/a
Post-quality control†	516,323	899,696	900,580	649,788	382,935

Supplementary Table 4: Details of the quality control filters applied to each genome-wide association study. Genotyped single nucleotide polymorphisms (SNPs) with a call rate < 95% were excluded as were those with a minor allele frequency (MAF)< 0.01 or displaying significant deviation from Hardy-Weinberg equilibrium (HWE) (i.e. $P < 10^{-5}$). GWAS, genome-wide association study; 1958BC, 1958 birth cohort; UKBS, UK blood service; NSHLG, National Study of Hodgkin Lymphoma Genetics.

*info score and plate association (see www.wtccc.org.uk for details)

† filters for QC were performed simultaneously so numbers for each criteria may not sum to total removed.

Locus	Nearest gene(s)	Strongest published SNP	Published histological subtype	Literature OR (95% CI)	Literature *P*-value	Strongest signal in discovery GWAS	Position (hg19, bp)	LD (r^2)	Subtype	Risk Allele	Current GWAS OR (95% CI)	Current GWAS *P*-value
2p16.1	*REL*	rs1432295[1]	cHL	1.22 (1.14-1.30)	1.91×10^{-8}	rs2420518	61054980	0.55	NSHL	C	1.29 (1.19-1.41)	4.49 × 10-^{9}
3p24.1	*EOMES*	rs3806624[2]	cHL	1.26 (1.18-1.34)	1.14×10^{-12}	rs3806624	27764623	NA	NSHL	G	1.31 (1.20-1.42)	5.89×10^{-10}
5q31.1	*IL13*	rs20541[7]	NSHL	1.84 (1.53-2.21)	5.00×10^{-11}	rs848	131996500	0.3	cHL	A	1.22 (1.14-1.31)	2.76×10^{-8}
6q23.3	*HBS1L-MYB*	rs7745098[2]	cHL	1.21 (1.14-1.29)	3.42×10^{-9}	rs9402684	135419305	0.98	NSHL	C	1.21 (1.11-1.32)	6.51×10^{-6}
8q24.21	*PVT1-MYC*	rs2019960[1]	cHL	1.33 (1.23-1.44)	1.97×10^{-13}	rs34748721	129195943	0.3	NSHL	G	1.70 (1.42-2.02)	3.86×10^{-9}
10p14	*GATA3*	rs501764[1]	cHL	1.25 (1.13-1.37)	7.05×10^{-8}	rs2388486	8099021	0.71	NSHL	T	1.58 (1.42-1.76)	4.32×10^{-17}
19p13.3	*TCF3*	rs1860661[7]	cHL	1.28 (1.17-1.30)	2.00×10^{-8}	rs2012125	1630341	0.34	cHL	A	1.16 (1.09-1.24)	8.92×10^{-6}

Supplementary Table 5: Strongest association signals at previously published risk loci. Shown for each region are the published single nucleotide polymorphism (SNPs), the SNP with the strongest association in the genome-wide association study (GWAS) discovery dataset, and the odds ratio (OR) and *P*-values associated with each, along with the linkage disequilibrium (LD) metrics between the SNPs. Meta-analysis for current GWAS was undertaken using the inverse-variance approach under a fixed effects model. cHL, classical Hodgkin lymphoma. bp, base-pair; NSHL, nodular sclerosis Hodgkin lymphoma; OR, odds ratio; CI, confidence interval.

	Risk allele	Discovery GWAS		UK Replication 1		Meta	OR (95% CI)	I^2	P_{het}
		P-value	OR (95% CI)	*P*-value	OR (95% CI)	*P*-value			
**2p25.1, rs6431936**	A								
cHL		1.93×10^{-4}	0.90 (0.85-0.95)	0.43	0.96 (0.87-1.06)	2.97×10^{-4}	0.91 (0.87-0.96)	65	0.04
NSHL		5.91×10^{-7}	0.82 (0.75-0.89)	0.43	0.96 (0.87-1.06)	5.62×10^{-5}	0.88 (0.82-0.93)	63	0.05
MCHL		0.40	0.96 (0.86-1.06)			0.40	0.96 (0.86-1.06)	70	0.07
**9p23, rs169730**	G								
cHL		4.83×10^{-3}	0.84 (0.75-0.95)	0.83	1.02 (0.84-1.24)	0.02	0.89 (0.80-0.98)	52	0.10
NSHL		8.17×10^{-7}	0.68 (0.52-0.88)	0.94	1.01 (0.83-1.22)	3.21×10^{-4}	0.79 (0.69-0.90)	78	0.00
MCHL		0.09	1.21 (0.97-1.51)			9.00×10^{-2}	1.21 (0.97-1.51)	0	0.32

Supplementary Table 6: Summary statistics for single nucleotide polymorphims demonstrating an association with nodular sclerosis Hodgkin lymphoma (NSHL) risk in the genome-wide association study meta-analysis ($P < 1.0 \times 10^{-7}$), but failed to replicate. Odds ratios (OR) derived with respect to the risk allele. Shown are discovery association *P*-values for individual studies (logistic regression) and from meta-analysis of the three GWAS datasets (inverse-variance approach under a fixed effects model). GWAS, genome-wide association study; cHL, classical Hodgkin lymphoma; MCHL, mixed cellularity Hodgkin lymphoma.

		3q28	6q22.33	6q23.3	10p14	13q34	16p13.13
		rs4459895	rs9482849	rs6928977	rs3781093	rs112998813	rs34972832
UK-NSHLG-GWAS Cases	AA	93/92	105/104	24/24	146/146	143/144	82/82
	Aa	53/54	49/50	75/75	33/34	21/20	38/38
	aa	15/15	6/6	67/67	2/1	2/2	4/4
	r^2	0.99	0.98	1.00	0.97	0.98	1.00

Supplementary Table 7: Concordance between imputed single nucleotide polymorphism and directly genotyped single nucleotide polymorphism. AA, major homozygote; AB, heterozygote; BB, minor homozygote. r^2 indicates Pearson product-moment correlation coefficient between imputed and genotyped single nucleotide polymorphism. NSHLG, National Study of Hodgkin Lymphoma Genetics.

	UK-GWAS		German-GWAS		UK-NSHLG-GWAS		Replication 1		Replication 2	
	Case	Control	Case	Control	Case	Control	Case	Control	Case	Control
**3q28, rs4459895**										
cHL	0.24	0.19	0.22	0.19	0.22	0.19	0.23	0.18	0.22	0.19
NSHL	0.25		0.22		0.23		0.23		0.23	
MCHL			0.20		0.18				0.19	
**6q22.33, rs9482849**										
cHL	0.20	0.16	0.17	0.14	0.18	0.15	0.17	0.16	0.17	0.15
NSHL	0.20		0.18		0.19		0.17		0.17	
MCHL			0.15		0.17					
**6q23.3, rs6928977**										
cHL	0.36	0.42	0.38	0.41	0.39	0.43	0.38	0.41	0.40	0.43
NSHL	0.35		0.35		0.37		0.39		0.37	
MCHL			0.40		0.41					
**10p14, rs3781093**										
cHL	0.10	0.14	0.12	0.15	0.11	0.14	0.11	0.14	0.11	0.11
NSHL	0.09		0.11		0.09		0.11		0.11	
MCHL			0.12		0.12				0.15	
**13q34, rs112998813**										
cHL	0.07	0.07	0.08	0.06	0.08	0.07	0.09	0.07	0.07	0.07
NSHL	0.09		0.08		0.10		0.09		0.08	
MCHL										
**16p13.13, rs34972832**										
cHL	0.22	0.19	0.24	0.19	0.20	0.19	0.23	0.20	0.22	0.20
NSHL	0.24		0.26		0.22		0.23		0.22	
MCHL			0.18		0.19		0.20		0.20	

Supplementary Table 8: Allele frequencies of newly identified classical Hodgkin lymphoma risk single nucleotide polymorphisms in different study series. GWAS, genome-wide association study; cHL, classical Hodgkin lymphoma; NSHLG, National Study of Hodgkin lymphoma Genetic; NSHL, nodular sclerosis Hodgkin lymphoma; MCHL, mixed cellularity Hodgkin lymphoma.

	Risk allele	Risk allele frequency	Meta-analysis (fixed effects)		Test of heterogeneity	
			P-value	OR (95% CI)	I^2 (95% CI)	P_{het}
**3q28, rs4459895**	A	0.20				
cHL			4.45×10^{-18}	1.30 (1.23-1.38)	13 (0-81)	0.33
NSHL			9.43×10^{-17}	1.39 (1.28-1.50)	0 (0-2)	0.93
MCHL			0.55	1.04 (0.92-1.19)	0 (0-46)	0.82
**6q22.33, rs9482849**	C	0.17				
cHL			1.52×10^{-8}	1.20 (1.13-1.28)	3 (0-80)	0.39
NSHL			4.13×10^{-6}	1.21 (1.12-1.33)	10 (0-81)	0.35
MCHL			0.16	1.10 (0.96-1.26)	0 (0)	0.97
**6q23.3, rs6928977**	G	0.57				
cHL			1.24×10^{-10}	1.17 (1.12-1.23)	0 (0-40)	0.85
NSHL			4.62×10^{-11}	1.23 (1.16-1.31)	26 (0-71)	0.25
MCHL			0.22	1.06 (0.96-1.17)	0 (0)	0.22
**10p14, rs3781093**	T	0.88				
cHL			4.91×10^{-12}	1.28 (1.19-1.37)	64 (32-88)	0.01
NSHL			9.49×10^{-13}	1.39 (1.28-1.53)	61 (0-85)	0.06
MCHL			0.16	0.91 (0.79-1.04)	73 (8-92)	0.03
**13q34, rs112998813**	C	0.08				
cHL			2.70×10^{-4}	1.19 (1.08-1.30)	13 (0-82)	0.32
NSHL			4.41×10^{-8}	1.39 (1.23-1.56)	27 (0-71)	0.24
MCHL			0.75	1.03 (0.85-1.25)	0 (0-82)	0.56
**16p13.13, rs34972832**	A	0.18				
cHL			8.03×10^{-7}	1.16 (1.09-1.23)	6 (0-80)	0.37
NSHL			2.12×10^{-8}	1.24 (1.15-1.34)	37 (0-76)	0.18
MCHL			0.70	0.98 (0.86-1.10)	0(0)	0.94

Supplementary Table 9: Results of fixed meta-analysis of newly identified classical Hodgkin lymphoma risk single nucleotide polymorphisms. OR, odds ratio; CI, confidence interval; cHL, classical Hodgkin lymphoma; NSHL, nodular sclerosis Hodgkin lymphoma; MCHL, mixed cellularity Hodgkin lymphoma; P_{het}, *P*-value for heterogeneity; I^2, proportion of the total variation due to heterogeneity.

		Sex[1]	Age[2]	EBV tumour status[3]
Locus	SNP		*P*-value	
3q28	rs4459895	0.01	0.42	0.90
6q22.33	rs9482849	0.67	0.38	0.45
6q23.3	rs6928977	0.26	0.07	0.03
10p14	rs3781093	0.70	0.01	0.20
13q34	rs112998813	0.15	0.90	0.30
16p13.13	rs34972832	0.54	0.03	0.53

Supplementary Table 10: Relationship between new Hodgkin lymphoma risk single nucleotide polymorphisms and sex of classical Hodgkin lymphoma cases, young adult nodular sclerosis Hodgkin lymphoma (16-35) and Epstein-Barr Virus tumour status. *P*-value calculated from logistic regression in each individual dataset followed by a standard meta-analysis where data from multiple studies was available. SNP, single nucleotide polymorphism; EBV, Epstein-Barr Virus.

[1] UK-GWAS (men=58, women=531), German-GWAS (men=348, women=528), UK-NSHLG-GWAS (men=712, women=900), UK replication 1 (men=655, women=629) and UK replication 2 (men=309, women=246).

[2] German-GWAS (young adult NSHL=151, other NSHL=251), UK-NSHLG-GWAS (young adult NSHL=261, other NSHL=459), UK replication 1 (young adult NSHL=561, other NSHL=655 and UK replication 2 (young adult NSHL=118, other NSHL=50).

[3] UK replication 2 phase data sets (EBV positive=246, EBV negative=550).

Dataset	Locus	Probe ID	Gene	SNP	SNP bp	A1	A2	Freq (A1)	b_{GWAS}	SE_{GWAS}	P_{GWAS}	b_{eqtl}	SE_{eqtl}	P_{eqtl}	b_{xy}	SE_{SMR}	P_{SMR}	P_{HEIDI}
MuTHER	6q23.3	ILMN_1791006	*AHI1*	rs12206850	135797808	T	C	0.67	0.21	0.046	3.80×10^{-6}	0.31	0.019	1.71×10^{-60}	0.68	0.153	8.63×10^{-6}	0.11
MuTHER	10p14	ILMN_2406656	*GATA3*	rs1149901	8094787	T	C	0.19	0.37	0.049	4.71×10^{-14}	-0.12	0.015	2.30×10^{-15}	-3.14	0.575	4.70×10^{-8}	0.10

Supplementary Table 11: Summary-data-based mendelian randomization analysis at Hodgkin lymphoma risk loci. Shown are summary-data-based mendelian randomization (SMR) results for risk loci that exceeded the threshold of $P_{SMR} < 5.49x10^{-4}$, as well as genome-wide association study (GWAS) and expression quantitative trait loci (eQTL) association statistics for the strongest associated *cis*-eQTL in lymphoblastoid cells. GWAS and eQTL effect betas (b) are with respect to allele A1. SE, standard error. Note: the SMR method calculates eQTL SNP, single nucleotide polymorphism; bp, base pair.

Conditioned variant(s)	Significant variant	Risk allele	*P*-value	OR (95% CI)	I^2	P_{het}
NSHL						
	rs9269081	A	1.74×10^{-39}	0.55 (0.50-0.60)	17	0.299
rs9269081	HLA-DPB1*03:01	Present	3.35×10^{-17}	0.53 (0.46-0.62)	9	0.3315
rs9269081, HLA-DPB1*03:01	Val86 HLA-DRB1	Present	3.52×10^{-13}	0.70 (0.63-0.77)	34	0.2208
MCHL						
	rs1633096	A	2.72×10^{-23}	0.56 (0.50-0.63)	34	0.2554
rs1633096	rs13196329	C	2.58×10^{-14}	0.17 (0.11-0.27)	0	0.648
rs1633096, rs13196329	Val86 HLA-DRB1	Present	7.10×10^{-9}	0.73 (0.66-0.81)	0	0.3706

Supplementary Table 12: HLA associations for nodular sclerosis Hodgkin lymphoma and mixed cellularity Hodgkin lymphoma. The risk allele is the allele corresponding to the estimated odds ratio. cHL, classical Hodgkin lymphoma; NSHL, nodular sclerosis Hodgkin lymphoma; MCHL, mixed cellularity Hodgkin lymphoma; OR, odds ratio; CI, confidence interval; P_{het}, *P*-value for heterogeneity; I^2, proportion of the total variation due to heterogeneity.

	RA[8]			SLE[9]			MS[10]			PBC[11]			UC[12]			Celiac disease[13]		
	r_g	SE	*P*-value	r_g	SE	*P*-value	r_g	SE	*P*-value	r_g	SE	*P*-value	r_g	SE	*P*-value	r_g	SE	*P*-value
cHL	-0.10	0.10	0.30	0.12	0.12	0.32	0.35	0.17	0.04	-0.01	0.13	0.93	-0.23	0.09	0.01	-0.19	0.16	0.22
NSHL	-0.15	0.09	0.07	0.14	0.09	0.15	0.24	0.17	0.16	-0.02	0.14	0.86	-0.13	0.10	0.18	-0.17	0.15	0.25
MCHL	0.06	0.19	0.75	0.36	0.45	0.42	0.41	0.32	0.19	0.00	0.38	1.00	-0.10	0.14	0.48	-0.05	0.22	0.82

Supplementary Table 13: Genetic correlation of classical Hodgkin lymphoma, nodular sclerosis Hodgkin lymphoma and mixed cellularity Hodgkin lymphoma with autoimmune diseases. cHL, classical Hodgkin lymphoma; NSHL, nodular sclerosis Hodgkin lymphoma; MCHL, mixed cellularity Hodgkin lymphoma; SE, standard error; RA, rheumatoid arthritis; SLE, systemic lupus erythematosus; MS multiple sclerosis; PBC, primary biliary cirrhosis; UC, ulcerative colitis; $r_{g,}$ genetic correlation.

Single nucleotide polymorphism	Conditions	Primer designation	Sequence
rs6431936	Std42	Kasp A1	GAAGGTGACCAAGTTCATGCTGTTCTCAAGGTTGATGCTCTGGT
		Kasp A2	GAAGGTCGGAGTCAACGGATTGTTCTCAAGGTTGATGCTCTGGC
		Kasp C	GGTCAAGGGAGAATGTGACCCAATA
rs4459895	Std42	Kasp A1	GAAGGTGACCAAGTTCATGCTCATAAATTTCATATGCACTGGGAACCAAT
		Kasp A2	GAAGGTCGGAGTCAACGGATTAAATTTCATATGCACTGGGAACCAAG
		Kasp C	CCACCGCAATAAAACAAATATTGCAGTGAA
rs9482849	Std 42 plus 5	Kasp A1	GAAGGTGACCAAGTTCATGCTATTAGTTCTGTCTCTCTAGAGAAC
		Kasp A2	GAAGGTCGGAGTCAACGGATTCCTATTAGTTCTGTCTCTCTAGAGAAT
		Kasp C	CTTAGAGTTGTCTCCTAGCCTTTTCTAAT
rs6928977	Std42	Kasp A1	GAAGGTGACCAAGTTCATGCTACTCTAAAAAAAAATGGCATTCCTTAAGG
		Kasp A2	GAAGGTCGGAGTCAACGGATTCACTCTAAAAAAAAATGGCATTCCTTAAGT
		Kasp C	GGGAATAGAGGAGTGTCAAGAATGAAAAT
rs169703	Std42	Kasp A1	GAAGGTGACCAAGTTCATGCTGAAGTTGTTCCCTTTGTGATACTCC
		Kasp A2	GAAGGTCGGAGTCAACGGATTGAAGTTGTTCCCTTTGTGATACTCG
		Kasp C	TCCTGCAGGTGCTCACACTCCT
rs3781093	Std42	Kasp A1	GAAGGTGACCAAGTTCATGCTGTCTCTCTACAGATCCTATAC
		Kasp A2	GAAGGTCGGAGTCAACGGATTCGCTGTCTCTCTACAGATCCTATAT
		Kasp C	CTCATCTCATCCCTAAAAGACAAGTGATT
rs112998813	Std 42 plus 5	Kasp A1	GAAGGTGACCAAGTTCATGCTACCAAAACCAAACAAAAAAG
		Kasp A2	GAAGGTCGGAGTCAACGGATTACCAAAACCAAACAAAAAAA
		Kasp C	AAAGAAAGACAGATGGCTCATGGCTATTT
rs34972832	Std42	Kasp A1	GAAGGTGACCAAGTTCATGCTGTTTAGCATGAGTAAATAAAATTGCCTAAT
		Kasp A2	GAAGGTCGGAGTCAACGGATTGTTTAGCATGAGTAAATAAAATTGCCTAAC
		Kasp C	AAATCTCAAGTATTCCATTTGGCTGCCT

Supplementary Table 14: Primer sequence for replication genotyping using competitive allele-specific PCR KASPar chemistry.

Std42

Hot Start: 94ºC for 15 minutes

Stage 1: 20 cycles

94ºC for 10 seconds
57ºC for 5 seconds
72ºC for 10 seconds

Stage 2: 22 cycles

94ºC for 10 seconds
57ºC for 20 seconds

Std42plus5

- Hot Start: 94ºC for 15 minutes
- Stage 1: 20 cycles
  - 94ºC for 10 seconds
  - 57ºC for 5 seconds
  - 72ºC for 10 seconds
- Stage 2: 22 cycles
  - 94ºC for 10 seconds
  - 57ºC for 20 seconds
  - 72ºC for 40 seconds
- Stage 3: 5 cycles
  - 94ºC for 10 seconds
  - 57ºC for 1 minute

REFERENCES

1. Enciso-Mora, V. *et al.* A genome-wide association study of Hodgkin's lymphoma identifies new susceptibility loci at 2p16.1 (REL), 8q24.21 and 10p14 (GATA3). *Nat Genet* **42**, 1126-1130 (2010).
2. Frampton, M. *et al.* Variation at 3p24.1 and 6q23.3 influences the risk of Hodgkin's lymphoma. *Nat Commun* **4**, 2549 (2013).
3. Penegar, S. *et al.* National study of colorectal cancer genetics. *Br J Cancer* **97**, 1305-9 (2007).
4. Matakidou, A. *et al.* Case-control study of familial lung cancer risks in UK women. *International Journal of Cancer* **116**, 445-450 (2005).
5. Lake, A. *et al.* Mutations of NFKBIA, encoding IkappaB alpha, are a recurrent finding in classical Hodgkin lymphoma but are not a unifying feature of non-EBV-associated cases. *Int J Cancer* **125**, 1334-42 (2009).
6. Hjalgrim, H. & Engels, E.A. Infectious aetiology of Hodgkin and non-Hodgkin lymphomas: a review of the epidemiological evidence. *J Intern Med* **264**, 537-48 (2008).
7. Cozen, W. *et al.* A meta-analysis of Hodgkin lymphoma reveals 19p13.3 TCF3 as a novel susceptibility locus. *Nat Commun* **5**(2014).
8. Okada, Y. *et al.* Genetics of rheumatoid arthritis contributes to biology and drug discovery. *Nature* **506**, 376-81 (2014).
9. Bentham, J. *et al.* Genetic association analyses implicate aberrant regulation of innate and adaptive immunity genes in the pathogenesis of systemic lupus erythematosus. *Nat Genet* **47**, 1457-1464 (2015).
10. Sawcer, S. *et al.* Genetic risk and a primary role for cell-mediated immune mechanisms in multiple sclerosis. *Nature* **476**, 214-9 (2011).
11. Cordell, H.J. *et al.* International genome-wide meta-analysis identifies new primary biliary cirrhosis risk loci and targetable pathogenic pathways. *Nat Commun* **6**, 8019 (2015).
12. Anderson, C.A. *et al.* Meta-analysis identifies 29 additional ulcerative colitis risk loci, increasing the number of confirmed associations to 47. *Nat Genet* **43**, 246-52 (2011).
13. Dubois, P.C.A. *et al.* Multiple common variants for celiac disease influencing immune gene expression. *Nat Genet* **42**, 295-302 (2010).
